# PAC-Bayes Bounds on Variational Tempered Posteriors for Markov Models

**DOI:** 10.3390/e23030313

**Published:** 2021-03-06

**Authors:** Imon Banerjee, Vinayak A. Rao, Harsha Honnappa

**Affiliations:** 1 Department of Statistics, Purdue University, West Lafayette, IN 47907, USA; ibanerj@purdue.edu; 2 School of Industrial Engineering, Purdue University, West Lafayette, IN 47907, USA; honnappa@purdue.edu

**Keywords:** ergodicity, Markov chain, probably approximately correct, variational Bayes

## Abstract

Datasets displaying temporal dependencies abound in science and engineering applications, with Markov models representing a simplified and popular view of the temporal dependence structure. In this paper, we consider Bayesian settings that place prior distributions over the parameters of the transition kernel of a Markov model, and seek to characterize the resulting, typically intractable, posterior distributions. We present a Probably Approximately Correct (PAC)-Bayesian analysis of variational Bayes (VB) approximations to tempered Bayesian posterior distributions, bounding the model risk of the VB approximations. Tempered posteriors are known to be robust to model misspecification, and their variational approximations do not suffer the usual problems of over confident approximations. Our results tie the risk bounds to the mixing and ergodic properties of the Markov data generating model. We illustrate the PAC-Bayes bounds through a number of example Markov models, and also consider the situation where the Markov model is misspecified.

## 1. Introduction

This paper presents probably approximately correct (PAC)-Bayesian bounds on variational Bayesian (VB) approximations of fractional or tempered posterior distributions for Markov data generation models. Exact computation of either standard or tempered posterior distributions is a hard problem that has, broadly speaking, spawned two classes of computational methods. The first, Markov chain Monte Carlo (MCMC), constructs ergodic Markov chains to approximately sample from the posterior distribution. MCMC is known to suffer from high variance and complex diagnostics, leading to the development of variational Bayesian (VB) [1] methods as an alternative in recent years. VB methods pose posterior computation as a variational optimization problem, approximating the posterior distribution of interest by the ‘closest’ element of an appropriately defined class of ‘simple’ probability measures. Typically, the measure of closeness used by VB methods is the Kullback–Leibler (KL) divergence. Excellent introductions to this so-called *KL-VB* method can be found in [2,3,4]. More recently, there has also been interest in alternative divergence measures, particularly the α-Rényi divergence [5,6,7], though in this paper, we focus on the KL-VB setting.

Theoretical properties of VB approximations, and in particular asymptotic frequentist consistency, have been studied extensively under the assumption of an independent and identically distributed (i.i.d.) data generation model [4,8,9]. On the other hand, the common setting where data sets display temporal dependencies presents unique challenges. In this paper, we focus on homogeneous Markov chains with parameterized transition kernels, representing a parsimonious class of data generation models with a wide range of applications. We work in the Bayesian framework, focusing on the posterior distribution over the unknown parameters of the transition kernel. Our theory develops PAC bounds that link the ergodic and mixing properties of the data generating Markov chain to the Bayes risk associated with approximate posterior distributions.

Frequentist consistency of Bayesian methods, in the sense of concentration of the posterior distribution around neighborhoods of the ‘true’ data generating distribution, have been established in significant generality, in both the i.i.d. [10,11,12] and in the non-i.i.d. data generation setting [13,14]. More recent work [14,15,16] has studied fractional or tempered posteriors, a class of generalized Bayesian posteriors obtained by combining the likelihood function raised to a fractional power with an appropriate prior distribution using Bayes’ theorem. Tempered posteriors are known to be robust against model misspecification: in the Markov setting we consider, the associated stationary distribution as well as mixing properties are sensitive to model parameterization. Further, tempered posteriors are known to be much simpler to analyze theoretically [14,16]. Therefore, following [14,15,16] we focus on tempered posterior distributions on the transition kernel parameters, and study the rate of concentration of variational approximations to the tempered posterior. Equivalently, as shown in [16] and discussed in Section 1.1, our results also apply to so-called α-variational approximations to standard posterior distributions over kernel parameters. The latter are modifications of the standard KL-VB algorithm to address the well-known problem of overconfident posterior approximations.

While there have been a number of recent papers studying the consistency of approximate variational posteriors [5,8,15] in the large sample limit, rates of convergence have received less attention. Exceptions include [9,15,17], where an i.i.d. data generation model is assumed. [15] establishes PAC-Bayes bounds on the convergence of a variational tempered posterior with fractional powers in the range [0, 1), while [9] considers the standard variational posterior case (where the fractional power equals 1). [17], on the other hand, establishes PAC-Bayes bounds for risk-sensitive Bayesian decision making problems in the standard variational posterior setting. The setting in [15] allows for model misspecification and the analysis is generally more straightforward than that in [9,17]. Our work extends [15] to the setting of a discrete-time Markov data generation model.

Our first results in Theorem 1 and Corollary 1 of Section 2 establish PAC-Bayes bounds for sequences with arbitrary temporal dependence. Our results generalize [15], [Theorem 2.4] to the non-i.i.d. data setting in a straightforward manner. Note that Theorem 1 also recovers ([16], [Theorem 3.3]), which is established under different ‘existence of test’ conditions. Our objective in this paper is to explicate how the ergodic and mixing properties of the Markov data generating process influences the PAC-Bayes bound. The sufficient conditions of our theorem, bounding the mean and variance of the log-likelihood ratio of the data, allows for developing this understanding, without the technicalities of proving the existence of test conditions intruding on the insights.

In Section 3, we study the setting where the data generating model is a stationary α-mixing Markov chain. Stationarity means that the Markov chain is initialized with the invariant distribution corresponding to the parameterized transition kernel, implying all subsequent states also follow this marginal distribution. The α-mixing condition ensures that the variance of the likelihood ratio of the Markov data does not grow faster than linear in the sample size. Our main results in this setting are applicable when the state space of the Markov chain is either continuous or discrete. The primary requirement on the class of data generating Markov models is for the log-likelihood ratio of the parameterized transition kernel and invariant distribution to satisfy a Lipschitz property. This condition implies a decoupling between the model parameters and the random samples, affording a straightforward verification of the mean and variance bounds. We highlight this main result by demonstrating that it is satisfied by a finite state Markov chain, a birth-death Markov chain on the positive integers, and a one-dimensional Gaussian linear model.

In practice, the assumption that the data generating model is stationary is unlikely to be satisfied. Typically, the initial distribution is arbitrary, with the state distribution of the Markov sequence converging weakly to the stationary distribution. In this setting, we must further assume that the class of data generating Markov chains are geometrically ergodic. We show that this implies the boundedness of the mean and variance of the log-likelihood ratio of the data generating Markov chain. Alternatively, in Theorem 4 we directly impose a drift condition on random variables that bound the log-likelihood ratio. Again, in this more general nonstationary setting, we illustrate the main results by showing that the PAC-Bayes bound is satisfied by a finite state Markov chain, a birth-death Markov chain on the positive integers, and a one-dimensional Gaussian linear model.

In preparation for our main technical results starting in Section 2 we first note relevant notations and definitions in the next section.

### 1.1. Notations and Definitions

We broadly adopt the notation in [15]. Let the sequence of random variables $X^{n}=\left(X_{0},\ldots ,X_{n}\right)\subset R^{m\times (n+1)}$ represent a dataset of n+1 observations drawn from a joint distribution $P_{\theta_{0}}^{\left(n\right)}$, where $\theta_{0}\in \Theta \subseteq R^{d}$ is the ‘true’ parameter underlying the data generation process. We assume the state space $S\subseteq R^{m}$ of the random variables $X_{i}$ is either discrete-valued or continuous, and write $\left\{x_{0},\cdots ,x_{n}\right\}$ for a realization of the dataset. We also adopt the convention that 0log(0/0)=0.

For each θ∈Θ, we will write $p_{\theta }^{\left(n\right)}$ as the probability density of $P_{\theta }^{\left(n\right)}$ with respect to some measure $Q^{\left(n\right)}$, i.e., $p_{\theta }^{\left(n\right)}:=\frac{dP_{\theta }^{\left(n\right)}}{dQ^{\left(n\right)}}$, where $Q^{\left(n\right)}$ is either Lebesgue measure or the counting measure. Unless stated otherwise, all probabilities, expectations and variances, which we represent as *P*, E[X] and Var[X], are with respect to the true distribution $P_{\theta_{0}}^{\left(n\right)}$.

Let π(θ) be a *prior* distribution with support Θ. The $\alpha^{te}$-*fractional posterior* is defined as

(1) $$\begin{array}{cc} \pi_{n,\alpha^{te}|X^{n}}\left(d\theta \right) & :=\frac{e^{-\alpha^{te}r_{n}\left(\theta ,\theta_{0}\right)\left(X^{n}\right)}\pi \left(d\theta \right)}{\int e^{-\alpha^{te}r_{n}\left(\theta ,\theta_{0}\right)\left(X^{n}\right)}\pi \left(d\theta \right)}, \end{array}$$

where, for $\theta_{0},\theta \in \Theta$, $r_{n}\left(\theta ,\theta_{0}\right)\left(\cdot \right):=\log\left(\frac{p_{\theta_{0}}^{\left(n\right)}\left(\cdot \right)}{p_{\theta }^{\left(n\right)}\left(\cdot \right)}\right)$, is the log-likelihood ratio of the corresponding density functions, and $\alpha^{te}\in \left(0,\infty \right)$ is a tempering coefficient. Setting $\alpha^{te}=1$ recovers the standard Bayesian posterior. Note that we will use superscripts to distinguish different quantities that are referred to just as α in the literature.

The *Kullback–Leibler* (KL) divergence between distributions P,Q is defined as

$$\begin{array}{c} K\left(P,Q\right):=\int_{X}\log\left(\frac{p(x)}{q(x)}\right)p\left(x\right)dx, \end{array}$$

where p,q are the densities corresponding to P,Q on some sample space X. In particular, the KL divergence between the distributions parameterized by $\theta_{0}$ and θ is

(2) $$\begin{array}{cc} K(P_{\theta_{0}}^{\left(n\right)},P_{\theta }^{\left(n\right)}) & :=\int \log\left(\frac{p_{\theta_{0}}^{\left(n\right)}\left(x_{0},\cdots ,x_{n}\right)}{p_{\theta }^{\left(n\right)}\left(x_{0},\cdots ,x_{n}\right)}\right)p_{\theta_{0}}^{\left(n\right)}\left(x_{0},\cdots ,x_{n}\right)dx_{0}\cdots dx_{n}\\ \; & =\int r_{n}\left(\theta ,\theta_{0}\right)\left(x_{0},\cdots ,x_{n}\right)p_{\theta_{0}}^{n}\left(x_{0},\cdots ,x_{n}\right)dx_{0}\cdots dx_{n}. \end{array}$$

The *$\alpha^{re}$-Rényi divergence*$D_{\alpha^{re}}\left(P_{\theta }^{\left(n\right)},P_{\theta_{0}}^{\left(n\right)}\right)$ is defined as

(3) $$\begin{array}{cc} D_{\alpha^{re}}\left(P_{\theta }^{\left(n\right)},P_{\theta_{0}}^{\left(n\right)}\right) & :=\frac{1}{\alpha^{re}-1}\log\int \exp\left(-\alpha^{re}r_{n}\left(\theta ,\theta_{0}\right)\left(x_{0},\cdots ,x_{n}\right)\right)p_{\theta_{0}}^{\left(n\right)}\left(x_{0},\cdots ,x_{n}\right)dx_{0}\cdots dx_{n}, \end{array}$$

where $\alpha^{re}\in \left(0,1\right)$. As $\alpha^{re}\to 1$, the $\alpha^{re}$-Rényi divergence recovers the KL divergence.

Let F be some class of distributions with support in $R^{d}$ and such that any distribution *P* in F is absolutely continuous with respect to the tempered posterior: $P\ll \pi_{n,\alpha^{te}|X^{n}}$.

Many choices of F exist; for instance (see also [15]), F can be the set of Gaussian measures, denoted $F_{id}^{\Phi }$:

(4) $$\begin{array}{cc} F_{id}^{\Phi } & =\{\Phi \left(d\theta ;\mu ,\Sigma \right):\mu \in R^{d},\Sigma_{d\times d}\in P.D.\}, \end{array}$$

where P.D. references the class of positive definite matrices. Alternately, F can be the family of *mean-field* or factored distributions where the components $\theta_{i}$ of θ are independent of each other. Let ${\tilde{\pi }}_{n,\alpha^{te}|X^{n}}$ be the variational approximation to the tempered posterior, defined as

(5) $$\begin{array}{cc} \;{\tilde{\pi }}_{n,\alpha^{te}|X^{n}} & :=\underset{\rho \in F}{\arg\min}\;K\left(\rho ,\pi_{n,\alpha^{te}|X^{n}}\right) \end{array}$$

It is easy to see that finding ${\tilde{\pi }}_{n,\alpha^{te}|X^{n}}$ in Equation (5) is equivalent to the following optimization problem:

(6) $$\begin{array}{cc} \;{\tilde{\pi }}_{n,\alpha^{te}|X^{n}} & :=\underset{\rho \in F}{\arg\max}\left[\int r_{n}\left(\theta ,\theta_{0}\right)\left(x_{0},\cdots ,x_{n}\right)\rho \left(d\theta \right)-{\left(\alpha^{te}\right)}^{-1}K\left(\rho ,\pi \right)\right]. \end{array}$$

Setting $\alpha^{te}=1$ again recovers the usual variational solution that seeks to approximate the posterior distribution with the closest element of F (the right-hand side above is called the evidence lower bound (ELBO)). Other settings of $\alpha^{te}$ constitute $\alpha^{te}$-variational inference [16], which seeks to regularize the ‘overconfident’ approximate posteriors that standard variational methods tend to produce.

Our results in this paper focus on parametrized Markov chains. We term a Markov chain as ‘parameterized’ if the transition kernel $p_{\theta }\left(\cdot |\cdot \right)$ is parametrized by some $\theta \in \Theta \subseteq R^{d}$. Let $q^{\left(0\right)}\left(\cdot \right)$ be the initial density (defined with respect to the Lebesgue measure over $R^{m}$) or initial probability mass function. Then, the joint density is $p_{\theta }^{\left(n\right)}\left(x_{0},\cdots ,x_{n}\right)=q^{\left(0\right)}\left(x_{0}\right)\prod_{i=0}^{n-1}p_{\theta }\left(x_{i+1}|x_{i}\right)$; recall, this joint density $p_{\theta }^{\left(n\right)}\left(x_{0},\cdots ,x_{n}\right)$ corresponds to the walk probability of a time-homogeneous Markov chain. We assume that corresponding to each transition kernel $p_{\theta },\;\theta \in \Theta ,$ there exists an invariant distribution $q_{\theta }^{\left(\infty \right)}\equiv q_{\theta }$ that satisfies

$$q_{\theta }\left(x\right)=\int p_{\theta }\left(x|y\right)q_{\theta }\left(dy\right)\;\forall x\in R^{m},\theta \in \Theta .$$

We will also use $q_{\theta }$ to designate the density of the invariant measure (as before, this is with respect to the Lebesgue or counting measure for continuous or discrete state spaces, respectively). A Markov chain is stationary if its initial distribution is the invariant probability distribution, that is, $X_{0}∼q_{\theta }$.

Our results in the ensuing sections will be established under strong mixing conditions [18] on the Markov chain. Specifically, recall the definition of the α-mixing coefficients of a Markov chain $\left\{X_{n}\right\}$:

**Definition** **1** (α-mixing coefficient). *Let $M_{i}^{j}$ denote the σ-field generated by the Markov chain $\left\{X_{k}:i\leq k\leq j\right\}$ parameterized by θ∈Θ. Then, the α-mixing coefficient is defined as*

(7) $$\begin{array}{c} \alpha_{k}=\underset{t>0}{\sup}\underset{\left(A,B\right)\in M_{-\infty }^{t}\times M_{t+k}^{\infty }}{\sup}\left|P_{\theta }\left(A\cap B\right)-P_{\theta }\left(A\right)P_{\theta }\left(B\right)\right|. \end{array}$$

Informally speaking, the α-mixing coefficients $\left\{\alpha_{k}\right\}$ measure the dependence between any two events *A* (in the ‘history’ σ-algebra) and *B* (in the ‘future’ σ-algebra) with a time lag *k*. We note that we do not use superscripts to identify these α parameters, since they are the only ones with subscripts, and can be identified through this.

## 2. A Concentration Bound for the $\alpha^{\mathrm{re}}$-Rényi Divergence

The object of analysis in what follows is the probability measure ${\tilde{\pi }}_{n,\alpha^{te}|X^{n}}\left(\theta \right)$, the variational approximation to the tempered posterior. Our main result establishes a bound on the Bayes risk of this distribution; in particular, given a sequence of loss functions $\ell_{n}\left(\theta ,\theta_{0}\right)$, we bound $\int \ell_{n}\left(\theta ,\theta_{0}\right){\tilde{\pi }}_{n,\alpha^{te}|X^{n}}\left(\theta \right)d\theta$. Following recent work in both the i.i.d. and dependent sequence settings [14,15,16], we will use $\ell_{n}\left(\theta ,\theta_{0}\right)=D_{\alpha^{re}}\left(P_{\theta }^{\left(n\right)},P_{\theta_{0}}^{\left(n\right)}\right)$, the $\alpha^{re}$-Rényi divergence between $P_{\theta }^{\left(n\right)}$ and $P_{\theta_{0}}^{\left(n\right)}$ as our loss function. Unlike loss functions like Euclidean distance, Rényi divergence compares θ and $\theta_{0}$ through their effect on observed sequences, so that issues like parameter identifiability no longer arise. Our first result generalizes [15], [Theorem 2.1] to a general non-i.i.d. data setting.

**Proposition** **1.** *Let F be a subset of all probability distributions on Θ. For any $\alpha^{re}\in \left(0,1\right)$, ϵ∈(0,1) and n≥1, the following probabilistic uniform upper bound on the expected $\alpha^{re}$-Rényi divergence holds:*

(8) $$\begin{array}{c} P\left[\underset{\rho \in F}{\sup}\int D_{\alpha^{re}}\left(P_{\theta }^{\left(n\right)},P_{\theta_{0}}^{\left(n\right)}\right)\rho \left(d\theta \right)\leq \frac{\alpha^{re}}{1-\alpha^{re}}\int r_{n}\left(\theta ,\theta_{0}\right)\rho \left(d\theta \right)+\frac{K\left(\rho ,\pi \right)+\log\left(\frac{1}{\epsilon }\right)}{1-\alpha^{re}}\right]\geq 1-\epsilon . \end{array}$$

The proof of Proposition 1 follows easily from [15], and we include it in Appendix B.1.1 for completeness. Mirroring the comments in [15], when $\rho ={\tilde{\pi }}_{n,\alpha^{te}}$ this result is precisely [14, Theorem 3.4]. We also note from [14] that $\forall \;\alpha^{re},\beta \in \left(0,1\right]$$\alpha^{re}$-Rényi divergences are all equivalent through the following inequality $\frac{\alpha^{re}\left(1-\beta \right)}{\beta (1-\alpha^{re})}D_{\beta }\leq D_{\alpha^{re}}\leq D_{\beta }\;\forall \;\alpha^{re}\leq \beta$. Hence, for the subsequent results, we simplify by assuming that $\alpha^{te}=\alpha^{re}$. This probabilistic bound implies the following PAC-Bayesian concentration bound on the model risk computed with respect to the fractional variational posterior:

**Theorem** **1.** *Let F be a subset of all probability distributions parameterized by Θ, and assume there exist $\epsilon_{n}>0$ and $\rho_{n}\in F$ such that*

*i.*  *$\int K\left(P_{\theta_{0}}^{\left(n\right)},P_{\theta }^{\left(n\right)}\right)\rho_{n}\left(d\theta \right)=\int E\left[r_{n}\left(\theta ,\theta_{0}\right)\right]\rho_{n}\left(d\theta \right)\leq n\epsilon_{n}$,*
*ii.*  *$\int \mathrm{Var}\left(r_{n}\left(\theta ,\theta_{0}\right)\right)\rho_{n}\left(d\theta \right)\leq n\epsilon_{n}$, and*
*iii.*  *$K\left(\rho_{n},\pi \right)\leq n\epsilon_{n}$.*

*Then, for any $\alpha^{re}\in \left(0,1\right)$ and (ϵ,η)∈(0,1)×(0,1),*

(9) $$\begin{array}{c} \;P\left[\int D_{\alpha^{re}}\left(P_{\theta }^{\left(n\right)},P_{\theta_{0}}^{\left(n\right)}\right){\tilde{\pi }}_{n,\alpha^{re}}\left(d\theta |X^{\left(n\right)}\right)\leq \frac{\left(\alpha^{re}+1\right)n\epsilon_{n}+\alpha^{re}\sqrt{\frac{n\epsilon_{n}}{\eta }}-\log\left(\epsilon \right)}{1-\alpha^{re}}\right]\geq 1-\epsilon -\eta . \end{array}$$

The proof of Theorem 1 is a generalization of [15] (Theorem 2.4) to the non-i.i.d. setting, and a special case of [16] (Theorem 3.1), where the problem setting includes latent variables. We include a proof for completeness. As noted in [15], the sufficient conditions follow closely from [13] and we will show that they hold for a variety of Markov chain models.

A direct corollary of Theorem 1 follows by setting $\eta =\frac{1}{n\epsilon_{n}}$, $\epsilon =e^{-n\epsilon_{n}}$ and using the fact that $e^{-n\epsilon_{n}}\geq \frac{1}{n\epsilon_{n}}$. Note that Equation (9) is vacuous if η+ϵ>1. Therefore, without loss of generality, we restrict ourselves to the condition $\frac{2}{n\epsilon_{n}}<1$.

**Corollary** **1.** *Assume $\exists \;\epsilon_{n}>0$, $\rho_{n}\in F$ such that the following conditions hold:*

*i.*  *$\int K\left(P_{\theta_{0}}^{\left(n\right)},P_{\theta }^{\left(n\right)}\right)\rho_{n}\left(d\theta \right)=\int E\left[r_{n}\left(\theta ,\theta_{0}\right)\right]\rho_{n}\left(d\theta \right)\leq n\epsilon_{n}$,*
*ii.*  *$\int \mathrm{Var}\left(r_{n}\left(\theta ,\theta_{0}\right)\right)\rho_{n}\left(d\theta \right)\leq n\epsilon_{n}$, and*
*iii.*  *$K\left(\rho_{n},\pi \right)\leq n\epsilon_{n}$.*

*Then, for any $\alpha^{re}\in \left(0,1\right)$,*

(10) $$\begin{array}{c} P\left[\int D_{\alpha^{re}}\left(P_{\theta }^{\left(n\right)},P_{\theta_{0}}^{\left(n\right)}\right){\tilde{\pi }}_{n,\alpha^{re}}\left(d\theta |X^{\left(n\right)}\right)\leq \frac{2\left(\alpha^{re}+1\right)\epsilon_{n}}{1-\alpha^{re}}\right]\geq 1-\frac{2}{n\epsilon_{n}}. \end{array}$$

We observe that Theorem 1 and Corollary 1 place no assumptions on the nature of the statistical dependence between data points. However, verification of the sufficient conditions is quite hard, in general. One of our key contributions is to verify that under reasonable assumptions on the smoothness of the transition kernel, the sufficient conditions of Theorem 1 and Corollary 1 are satisfied by ergodic Markov chains.

Observe that the first two conditions in Corollary 1 ensure that the distribution $\rho_{n}$ concentrates on parameters θ∈Θ around the true parameter $\theta_{0}$, while the third condition requires that $\rho_{n}$ not diverge from the prior π rapidly as a function of the sample size *n*. In general, verifying the first and third conditions is relatively straightforward. The second condition, on the other hand, is significantly more complicated in the current setting of dependent data, as the variance of $r_{n}\left(\theta ,\theta_{0}\right)$ includes correlations between the observations $\left\{X_{0},\cdots ,X_{n}\right\}$. In the next section, we will make assumptions on the transition kernels (and corresponding invariant densities) that ’decouple’ the temporal correlations and the model parameters in the setting of strongly mixing and ergodic Markov chain models, and allow for the verification of the conditions in Corollary 1. Towards this, Propositions 2 and 3 below characterize the expectation and variance of the log-likelihood ratio $r_{n}\left(\cdot ,\cdot \right)$ in terms of the one-step transition kernels of the Markov chain. First, consider the expectation of $r_{n}\left(\cdot ,\cdot \right)$ in condition (i).

**Proposition** **2.** *Fix $\theta_{1},\theta_{2}\in \Theta$ and consider the parameterized Markov transition kernels $p_{\theta_{1}}$ and $p_{\theta_{2}}$, and initial distributions $q_{\theta_{1}}^{\left(0\right)}$ and $q_{\theta_{2}}^{\left(0\right)}$. Let $p_{\theta_{1}}^{\left(n\right)}$ and $p_{\theta_{2}}^{\left(n\right)}$ be the corresponding joint probability densities; that is,*

(11) $$p_{\theta_{j}}^{\left(n\right)}\left(x_{0},\ldots ,x_{n}\right)=q_{\theta_{j}}^{\left(0\right)}\left(x_{0}\right)\prod_{i=1}^{n}p_{\theta_{i}}\left(x_{i}|x_{i-1}\right)$$

*for j∈{1,2}. Then, for any n≥1, the log-likelihood ratio $r_{n}\left(\theta_{2},\theta_{1}\right)$ satisfies*

(12) $$\begin{array}{cc} \;E_{\theta_{1}}\left[r_{n}\left(\theta_{2},\theta_{1}\right)\right]\; & =\sum_{i=1}^{n}E_{\theta_{1}}\left[\log\left(\frac{p_{\theta_{1}}\left(X_{i}|X_{i-1}\right)}{p_{\theta_{2}}\left(X_{i}|X_{i-1}\right)}\right)\right]+E_{\theta_{1}}\left[Z_{0}\right], \end{array}$$

*where $Z_{0}:=\log\left(\frac{q_{\theta_{1}}^{\left(0\right)}\left(X_{0}\right)}{q_{\theta_{2}}^{\left(0\right)}\left(X_{0}\right)}\right)$. The expectation in the first term is with respect to the joint density function $p_{\theta_{1}}\left(y,x\right)=p_{\theta_{1}}\left(y|x\right)q_{\theta_{1}}^{\left(i-1\right)}\left(x\right)$ where the marginal density satisfies*

$$\begin{array}{c} q_{\theta_{1}}^{\left(i-1\right)}\left(x\right)=\left\{\begin{array}{cc} \int p_{\theta_{1}}^{\left(i-1\right)}\left(x_{0},\ldots ,x_{i-2},x\right)dx_{0}\cdots dx_{i-2} & \;for\;i>1,and\\ q_{\theta_{1}}^{\left(0\right)}\left(x\right) & \;for\;i=1. \end{array}\right. \end{array}$$

*If the Markov chain is also stationary under $\theta_{1}$, then Equation (12) simplifies to*

(13) $$\begin{array}{c} E_{\theta_{1}}\left[r_{n}\left(\theta_{2},\theta_{1}\right)\right]=nE_{\theta_{1}}\left[\log\left(\frac{p_{\theta_{1}}\left(X_{1}|X_{0}\right)}{p_{\theta_{2}}\left(X_{1}|X_{0}\right)}\right)\right]+E_{\theta_{1}}\left[Z_{0}\right]. \end{array}$$

Notice that $E_{\theta_{1}}\left[r_{n}\left(\theta_{2},\theta_{1}\right)\right]$ is precisely the KL divergence, $K(P_{\theta_{1}}^{\left(n\right)},P_{\theta_{2}}^{\left(n\right)})$. Next, the following proposition uses [19] (Lemma 1.3) to upper bound the variance of the log-likelihood ratio.

**Proposition** **3.** *Fix $\theta_{1},\theta_{2}\in \Theta$ and consider parameterized Markov transition kernels $p_{\theta_{1}}$ and $p_{\theta_{2}}$, with initial distributions $q_{\theta_{1}}^{\left(0\right)}$ and $q_{\theta_{2}}^{\left(0\right)}$. Let $p_{\theta_{1}}^{\left(n\right)}$ and $p_{\theta_{2}}^{\left(n\right)}$ be the corresponding joint probability densities of the sequence $\left(x_{0},\cdots ,x_{n}\right)$, and $q_{\theta_{j}}^{\left(i\right)}$ the marginal density for i∈{1,…,n} and j∈{1,2}. Fix δ>0 and, for each i∈{1,⋯,n}, define*

$$\begin{array}{cc} C_{\theta_{1},\theta_{2}}^{\left(i\right)} & :=\int {\left|\log\left(\frac{p_{\theta_{1}}\left(x_{i}|x_{i-1}\right)}{p_{\theta_{2}}\left(x_{i}|x_{i-1}\right)}\right)\right|}^{2+\delta }p_{\theta_{1}}\left(x_{i}|x_{i-1}\right)q_{\theta_{1}}^{\left(i-1\right)}\left(x_{i-1}\right)dx_{i}dx_{i-1}. \end{array}$$

*Similarly, define $Z_{0}:=\log\left(\frac{q_{\theta_{1}}^{\left(0\right)}\left(X_{0}\right)}{q_{\theta_{2}}^{\left(0\right)}\left(X_{0}\right)}\right)$, and $D_{1,2}:=E_{\theta_{1}}{\left|Z_{0}\right|}^{2+\delta }.$ Suppose the Markov chain corresponding to $\theta_{1}$ is α-mixing with coefficients $\left\{\alpha_{k}\right\}$. Then,*

Varrn(θ1,θ2)<∑i,j=1n4n+2nδ/2(Cθ1,θ2(i)+Cθ1,θ2(j)+Cθ1,θ2(i)Cθ1,θ2(j))α|i−j|−1δ/(2+δ)(14)+∑i=1n4n+2nδ/2(Cθ1,θ2(i)+D1,2+Cθ1,θ2(i)D1,2)αi−1δ/(2+δ)(15)+Cov(Z0,Z0).

Note that this result holds for any parameterized Markov chain. In particular, when the Markov chain is stationary, $C_{\theta_{1},\theta_{2}}^{\left(i\right)}=C_{\theta_{1},\theta_{2}}^{\left(1\right)}\;\forall \;i$ and ∀θ∈Θ, and Equation (14) simplifies to

(16) $$\begin{array}{cc} \mathrm{Var}\left(r_{n}\left(\theta_{1},\theta_{2}\right)\right) & <n\left(\frac{4}{n}+6n^{\delta /2}C_{\theta_{1},\theta_{2}}^{\left(1\right)}\right)\left(\sum_{k\geq 0}\alpha_{k}^{\delta /(2+\delta )}\right)\\ \; & \;+\left(\frac{4}{n}+2n^{\delta /2}\left(C_{\theta_{1},\theta_{2}}^{\left(1\right)}+D_{1,2}+\sqrt{C_{\theta_{1},\theta_{2}}^{\left(1\right)}D_{1,2}}\right)\right)\left(\sum_{k\geq 1}\alpha_{k}^{\delta /(2+\delta )}\right)\\ \; & \;+\mathrm{Cov}(Z_{0},Z_{0}). \end{array}$$

If the sum $\sum_{k\geq 0}\alpha_{k}^{\delta /(2+\delta )}$ is infinite, the bound is trivially true. For it to be finite, of course, the coefficients $\alpha_{k}$ must decay to zero sufficiently quickly. For instance, Theorem A.1.2 shows that if the Markov chain is geometrically ergodic, then the α-mixing coefficients are geometrically decreasing. We will use this fact when the Markov chain is non-stationary, as in Section 4. In the next section, however, we first consider the simpler stationary Markov chain setting where geometric ergodic conditions are not explicitly imposed. We also note that unless only a finite number of $\alpha_{k}$ are nonzero, the sum $\sum_{k\geq 0}\alpha_{k}^{\delta /(2+\delta )}$ is infinite when δ=0, and our results will typically require δ>0.

## 3. Stationary Markov Data-Generating Models

Observe that the PAC-Bayesian concentration bound in Corollary 1 specifically requires bounding the mean and variance of the log-likelihood ratio $r_{n}\left(\theta ,\theta_{0}\right)$. We ensure this by imposing regularity conditions on the log-ratio of the one-step transition kernels and the corresponding invariant densities. Specifically, we assume the following conditions that decouple the model parameters from the random samples, allowing us to verify the bounds in Corollary 1.

**Assumption** **1.** *There exist positive functions $M_{k}^{\left(1\right)}\left(\cdot ,\cdot \right)$ and $M_{k}^{\left(2\right)}\left(\cdot \right)$, k∈{1,2,⋯,m} such that for any parameters $\theta_{1},\theta_{2}\in \Theta$, the log of the ratio of one-step transition kernels and the log of the ratio of the invariant distributions satisfy, respectively,*

(17) $$\begin{array}{c} \;|\log p_{\theta_{1}}\left(x_{1}|x_{0}\right)-\log p_{\theta_{2}}\left(x_{1}|x_{0}\right)|\leq \sum_{k=1}^{m}M_{k}^{\left(1\right)}\left(x_{1},x_{0}\right)\left|f_{k}^{\left(1\right)}\left(\theta_{2},\theta_{1}\right)\right|\;\forall \;\left(x_{0},x_{1}\right),\;and\; \end{array}$$

(18) $$\begin{array}{c} \;|\log q_{\theta_{1}}\left(x\right)-\log q_{\theta_{2}}\left(x\right)|\leq \sum_{k=1}^{m}M_{k}^{\left(2\right)}\left(x\right)\left|f_{k}^{\left(2\right)}\left(\theta_{2},\theta_{1}\right)\right|\;\forall \;x. \end{array}$$

*We further assume that for some δ>0, the functions $f_{k}^{\left(1\right)},f_{k}^{\left(2\right)}$ and $M_{k}^{\left(1\right)}$ satisfy the following:*

*i*  *there exist constants $C_{k}^{\left(t\right)}$ and measures $\rho_{n}\in F$ such that $\int |f_{k}^{\left(t\right)}\left(\theta ,\theta_{0}\right){|}^{2+\delta }\rho_{n}\left(d\theta \right)<\frac{C_{k}^{\left(t\right)}}{n}$ for t∈{1,2}, n≥1 and k∈{1,2,⋯,m}, and*
*ii*  *there exists a constant B such that $\int M_{k}^{\left(1\right)}{\left(x_{1},x_{0}\right)}^{2+\delta }p_{\theta_{j}}\left(x_{1}|x_{0}\right)q_{\theta_{j}}^{\left(0\right)}\left(x_{0}\right)dx_{1}dx_{0}<B,\;k\in \left\{1,\cdots ,m\right\}$ and j∈{1,2}.*

The following examples illustrate Equations (17) and (18) for discrete and continuous state Markov chains.

**Example** **1.** *Suppose $\left\{X_{0},\cdots ,X_{n}\right\}$ is generated by the birth-death chain with parameterized transition probability mass function,*

$$\begin{array}{c} p_{\theta }\left(j|i\right)=\left\{\begin{array}{cc} \theta & \;if\;j=i-1,\\ 1-\theta & \;if\;j=i+1. \end{array}\right. \end{array}$$

*In this example, the parameter θ denotes the probability of birth. We shall see that, m=3: $M_{1}^{\left(1\right)}\left(X_{1},X_{0}\right)=I_{\left[X_{1}=X_{0}+1\right]}$, $M_{2}^{\left(1\right)}\left(X_{1},X_{0}\right)=I_{\left[X_{1}=X_{0}-1\right]}$, and $M_{3}^{\left(1\right)}\left(X_{1},X_{0}\right)=1$. We also define $M_{1}^{\left(2\right)}\left(X_{0}\right)=1$, and set $M_{2}^{\left(2\right)}\left(X_{0}\right)$ and $M_{3}^{\left(2\right)}\left(X_{0}\right)$ both to $X_{0}-1$. Let $f_{1}^{\left(1\right)}\left(\theta ,\theta_{0}\right)=\log\left[\frac{\theta_{0}}{\theta }\right]$, $f_{2}^{\left(1\right)}\left(\theta ,\theta_{0}\right)=\log\left[\frac{1-\theta_{0}}{1-\theta }\right]$, $f_{3}^{\left(1\right)}\left(\theta ,\theta_{0}\right)=0$, $f_{1}^{\left(2\right)}\left(\theta ,\theta_{0}\right)=-f_{3}^{\left(2\right)}\left(\theta ,\theta_{0}\right)=\log\left[\frac{1-\theta_{0}}{1-\theta }\right]$, and $f_{2}^{\left(2\right)}\left(\theta ,\theta_{0}\right)=\log\left[\frac{\theta_{0}}{\theta }\right]$. The derivation of these terms and that they satisfy the conditions of Assumption 1 is provided in the proof of Proposition 6.*

**Example** **2.** *Suppose $\left\{X_{0},\cdots ,X_{n}\right\}$ is generated by the ‘simple linear’ Gauss–Markov model*

$$X_{n}=\theta X_{n-1}+W_{n},$$

*where $\left\{W_{n}\right\}$ is a sequence of i.i.d. standard Gaussian random variables. Then, m=2, with $M_{1}^{\left(1\right)}\left(X_{n},X_{n-1}\right)=\left|X_{n}X_{n-1}\right|$, $M_{2}^{\left(1\right)}\left(X_{n},X_{n-1}\right)=X_{n}^{2},M_{1}^{\left(2\right)}\left(x\right)=\frac{x^{2}}{2}$ and $M_{2}^{\left(2\right)}\left(X\right)=0$. Corresponding to these, we have $f_{1}^{\left(1\right)}\left(\theta ,\theta_{0}\right)=\left(\theta -\theta_{0}\right),f_{2}^{\left(1\right)}\left(\theta ,\theta_{0}\right)=\left(\theta_{0}^{2}-\theta^{2}\right),f_{1}^{\left(2\right)}\left(\theta_{0},\theta_{0}\right)=\left(\theta_{0}^{2}-\theta^{2}\right)$ and $f_{2}^{\left(2\right)}\left(\theta_{0},\theta_{0}\right)=0$. The derivation of these quantities and that these satisfy the conditions of Assumption 1 under appropriate choice of $\rho_{n}$ is shown in the proof of Proposition 10.*

Note that assuming the same number *m* of $M_{k}^{\left(1\right)}$ and $M_{k}^{\left(2\right)}$ involves no loss of generality, since these functions can be set to 0. Both Equations (17) and (18) can be viewed as generalized Lipschitz-smoothness conditions, recovering the usual Lipschitz-smoothness when m=1 and when $f_{k}^{\left(t\right)}$ is Euclidean distance. Our generalized conditions are useful for distributions like the Gaussian, where Lipschitz smoothness does not apply. From Jensen’s inequality we have $\int |f_{k}^{\left(t\right)}\left(\theta ,\theta_{0}\right)\left|\rho_{n}\left(d\theta \right)\right|\leq {\left[\int |f_{k}^{\left(t\right)}\left(\theta ,\theta_{0}\right){|}^{2+\delta }\rho_{n}\left(d\theta \right)\right]}^{\frac{1}{2+\delta }}$, and Assumption 1(i) above implies that for some constant C>0 and k∈{1,2,⋯,m},t∈{1,2},

(19) $$\begin{array}{cc} \int |f_{k}^{\left(t\right)}\left(\theta ,\theta_{0}\right)|\rho_{n}\left(d\theta \right) & \leq \frac{C}{n^{1/(2+\delta )}}<\frac{C}{\sqrt{n}}. \end{array}$$

Assumption 1(i) is satisfied in a variety of scenarios, for example, under mild assumptions on the partial derivatives of the functions $f_{k}^{\left(t\right)}$. To illustrate this, we present the following proposition.

**Proposition** **4.** *Let $f(\theta ,\theta_{0})$ be a function on a bounded domain with bounded partial derivatives with $f(\theta_{0},\theta_{0})=0$. Let $\left\{\rho_{n}\left(\cdot \right)\right\}$ be a sequence of probability densities on θ such that $E_{\rho_{n}}\left[\theta \right]=\theta_{0}$ and ${\mathrm{Var}}_{\rho_{n}}\left[\theta \right]=\frac{\sigma^{2}}{n}$ for some σ>0. Then, for some C>0,*

(20) $$\int |f\left(\theta ,\theta_{0}\right){|}^{2+\delta }\rho_{n}\left(d\theta \right)<\frac{C}{n}.$$

**Proof.**  Define $\partial_{\theta }f\left(\theta ,\theta_{0}\right):=\frac{\partial f(\theta ,\theta_{0})}{\partial \theta }$ as the partial derivative of the function *f*. By the mean value theorem, $|f\left(\theta ,\theta_{0}\right)|=|\theta -\theta_{0}\left|\right|\partial_{\theta }f\left(\theta^{*},\theta_{0}\right)|$, for some $\theta^{*}\in \left[\min\left\{\theta ,\theta_{0}\right\},\max\left\{\theta ,\theta_{0}\right\}\right]$. Since the partial derivatives are bounded, there exists L∈R such that $\partial_{\theta }f\left(\theta^{*},\theta_{0}\right)<L$, and $\int |f\left(\theta ,\theta_{0}\right){|}^{2+\delta }\rho_{n}\left(d\theta \right)<L^{2+\delta }\int {\left|\theta -\theta_{0}\right|}^{2+\delta }\rho_{n}\left(d\theta \right)$. Choose G>0 be such that |θ|<G, then ${\left|\frac{\theta -\theta_{0}}{2G}\right|}^{2+\delta }<{\left|\frac{\theta -\theta_{0}}{2G}\right|}^{2}$. Therefore, $\int |\theta -\theta_{0}{|}^{2+\delta }\rho_{n}\left(d\theta \right)<{\left(2G\right)}^{2+\delta }\mathrm{Var}\left[\frac{\theta }{2G}\right]<{\left(2G\right)}^{\delta }\frac{\sigma^{2}}{n}$. Now choosing ${\left(2G\right)}^{\delta }\sigma^{2}$ as *C* completes the proof. □

If $\partial_{\theta }f_{k}^{\left(t\right)}$ is continuous and Θ is compact, then $\partial_{\theta }f_{k}^{\left(t\right)}$ is always bounded. Furthermore, observe that if $E\left[M_{k}^{\left(1\right)}{\left(X_{1},X_{0}\right)}^{2+\delta }\right]<B$, without loss of generality we can use Jensen’s inequality to conclude that, for all 0<a<2+δ, $E\left[M_{k}^{\left(1\right)}{\left(X_{1},X_{0}\right)}^{a}\right]<B^{\frac{a}{2+\delta }}<B$.

We can now state the main theorem of this section.

**Theorem** **2.** *Let $\left\{X_{0},\ldots ,X_{n}\right\}$ be generated by a stationary, α-mixing Markov chain parametrized by $\theta_{0}\in \Theta$. Suppose that Assumption 1 holds and that the α-mixing coefficients satisfy $\sum_{k\geq 1}\alpha_{k}^{\delta /(2+\delta )}<+\infty$. Furthermore, assume that $K\left(\rho_{n},\pi \right)\leq \sqrt{n}C$ for some constant C>0. Then, the conditions of Corollary 1 are satisfied with $\epsilon_{n}=O\left(\max(\frac{1}{\sqrt{n}},\frac{n^{\delta /2}}{n})\right)$.*

Theorem 2 is satisfied by a large class of Markov chains, including chains with countable and continuous state spaces. In particular, if the Markov chain is geometrically ergodic, then it follows from Equation (A4) (in the appendix) that $\sum_{k\geq 1}\alpha_{k}^{\delta /(2+\delta )}<+\infty$. Observe that in order to achieve $O(\frac{1}{\sqrt{n}})$ convergence, we need δ≤1. Key to the proof of Theorem 2 is the fact that the variance of the log-likelihood ratio can be controlled via the application of Assumption 1 and Proposition 3. Note also that as δ decreases, satisfying the condition $\sum_{k\geq 1}\alpha_{k}^{\delta /(2+\delta )}$ requires the Markov chain to be faster mixing.

We now illustrate Theorem 2 for a number of Markov chain models. First, consider a birth-death Markov chain on a finite state space.

**Proposition** **5.** *Suppose the data-generating process is a birth-death Markov chain, with one-step transition kernel parametrized by the birth probability $\theta_{0}\in \Theta$. Let F be the set of all Beta distributions. We choose the prior to be a Beta distribution. Then, the conditions of Theorem 2 are satisfied and $\epsilon_{n}=O\left(\frac{1}{\sqrt{n}}\right)$.*

**Proof.**  The proof of Proposition 5 follows from the more general Proposition 8, by fixing the initial distribution to the invariant distribution under $\theta_{0}$. Therefore it has been omitted. We simply refer to the proof of Proposition 8 under a more general setup in Appendix B.3. □

The birth-death chain on the finite state space is, of course, geometrically ergodic and the α-mixing coefficients $\alpha_{k}$ decay geometrically. Note that the invariant distribution of this Markov chain is uniform over the state space, and consequently this is a particularly simple example. A more complicated and more realistic example is a birth-death Markov chain on the nonnegative integers. We note that if the probability of birth θ in a birth-death Markov chain on positive integers is greater than 0.5, then the Markov chain is transient, and consequently, not ergodic. Hence, our prior should be chosen to have support within (0,0.5). For that purpose, we define the class of scaled beta distributions.

**Definition** **2** (Scaled Beta). *If X is a beta distribution on with parameters a and b, then Y is said to be a scaled beta distribution with same parameters on the interval (c,m+c) if*

$$\begin{array}{cc} Y & =mx+c\;;\;\left(m,c\right)\in R^{2} \end{array}$$

*and in that case, the pdf of Y is obtained as*

$$\begin{array}{c} f\left(y\right)=\left\{\begin{array}{cc} \frac{1}{m\mathrm{Beta}(a,b)}{\left(\frac{y-c}{m}\right)}^{a-1}{\left(1-\frac{y-c}{m}\right)}^{b-1} & \mathrm{if}\;y\in (c,m+c),\\ \;0 & \mathrm{otherwise}. \end{array}\right.. \end{array}$$

Here, $E\left[Y\right]=m\frac{a}{a+b}+c$ and $\mathrm{Var}\left[Y\right]=m^{2}\frac{ab}{{\left(a+b\right)}^{2}\left(a+b+1\right)}$. For the birth-death chain, we set m=0.5 and c=0 giving it support on $\left(0,\frac{1}{2}\right)$. Setting m=2 and c=−1 gives a beta distribution rescaled to have support on (−1,1).

**Proposition** **6.** *Suppose the data-generating process is a positive recurrent birth-death Markov chain on the positive integers parameterized by the birth probability $\theta_{0}\in \left(0,\frac{1}{2}\right)$. Further let F be the set of all Beta distributions rescaled to have support $\left(0,\frac{1}{2}\right)$. We choose the prior to be a scaled Beta distribution on (0,1/2) with parameters a and b. Then, the conditions of Theorem 2 are satisfied with $\epsilon_{n}=O\left(\frac{1}{\sqrt{n}}\right)$.*

**Proof.**  The proof of Proposition 6 (for the stationary case) follows from the more general Proposition 9 (the nonstationary case) by fixing the initial distribution to the invariant distribution under $\theta_{0}$. We omit the proof and simply refer to the proof of Proposition 9 under a more general setup in Appendix B.3. □

Unlike with the finite state-space, the invariant distribution now depends on the parameter θ∈Θ, and verification of the conditions of the proposition is more involved. In Appendix A.2, we prove that the class of scaled beta distributions satisfy the condition $K\left(\rho_{n},\pi \right)\leq n\epsilon_{n}$ when the prior π is a beta or an uniform distribution. This fact will allow us to prove the above propositions.

Both Proposition 5 and Proposition 6 assume a discrete state space. The next example considers a strictly stationary simple linear model (as defined in Example 2), which has a continuous, unbounded state space.

**Proposition** **7.** *Suppose the data-generating model is a stationary simple linear model:*

(21) $$\begin{array}{cc} X_{n} & =\theta_{0}X_{n-1}+W_{n}, \end{array}$$

*where $\left\{W_{n}\right\}$ are i.i.d. standard Gaussian random variables and $|\theta_{0}|<1$. Suppose that F is the class of all beta distributions rescaled to have the support (−1,1). Then, the conditions of Theorem 2 are satisfied with $\epsilon_{n}=O\left(\frac{1}{\sqrt{n}}\right)$.*

**Proof.**  This is a special case of the more general non-stationary simple linear model which is detailed in Proposition 10. Therefore, the proof of the fact that the simple linear model satisfies Assumption 1 when starting from stationarity is deferred to the proof of Proposition 10. The simple linear model with $|\theta_{0}|<1$ has geometrically decreasing (and therefore summable) α-mixing coefficients as a consequence of [20] (eq. (15.49)) and Theorem A.1.2. Combining these two facts, it follows that the conditions of Theorem 2 are satisfied. □

Observe that Theorem 1 (and Corollary 1) are general, and hold for *any* dependent data-generating process. Therefore, there can be Markov chains that satisfy these, but do not satisfy Assumption 1 which entails some loss of generality. However, as our examples demonstrate, common Markov chain models do indeed satisfy the latter assumption.

## 4. Non-Stationary, Ergodic Markov Data-Generating Models

We call a time-homogeneous Markov chain *non-stationary* if the initial distribution $q^{\left(0\right)}$ is not the invariant distribution. There are two sets of results in this setting: in Theorem 3 and Theorem 4 we explicitly impose the α-mixing condition, while in Theorem 5 we impose a *f*-geometric ergodicity condition (Definition A.1.2 in the appendix). As seen in Equation (A4) (in the appendix) if the Markov chain is also geometrically ergodic, then ∀δ>0, $\sum \alpha_{k}^{\delta /(2+\delta )}<\infty$. This condition can be relaxed, albeit at the risk of more complicated calculations that, nonetheless, mirror those in the geometrically ergodic setting. A common thread through these results is that we must impose some integrability or regularity conditions on the functions $M_{k}^{\left(1\right)}$.

First, in Theorem 3 we assume that the $M_{k}^{\left(1\right)}$ functions in Assumption 1 are uniformly bounded and that the α-mixing condition is satisfied. This result holds for both discrete and continuous state space settings.

**Theorem** **3.** *Let $\left\{X_{0},\cdots ,X_{n}\right\}$ be generated by an α-mixing Markov chain parametrized by $\theta_{0}\in \Theta$ with transition probabilities satisfying Assumption 1 and with known initial distribution $q^{\left(0\right)}$. Let $\left\{\alpha_{k}\right\}$ be the α-mixing coefficients under $\theta_{0}$, and assume that $\sum_{k\geq 1}\alpha_{k}^{\delta /\left(2+\delta \right)}<+\infty$. Suppose that there exists B∈R such that $\sup_{x,y}\left|M_{k}^{\left(1\right)}\left(x,y\right)\right|<B$ for all k∈{1,2,⋯,m} in Assumption 1. Furthermore, assume that there exists $\rho_{n}\in F$ such that $K\left(\rho_{n},\pi \right)\leq \sqrt{n}C$ for some constant C>0. If the initial distribution $q^{\left(0\right)}$ satisfies $E_{q^{\left(0\right)}}{\left|M_{k}^{\left(2\right)}\left(X_{0}\right)\right|}^{2}<+\infty$ for all k∈{1,2,⋯,m}, then the conditions of Corollary 1 are satisfied with $\epsilon_{n}=O\left(\max(\frac{1}{\sqrt{n}},\frac{n^{\delta /2}}{n})\right)$.*

The following result in Proposition 8 illustrates Theorem 3 in the setting of a finite state birth-death Markov chain.

**Proposition** **8.** *Suppose the data-generating process is a finite state birth-death Markov chain, with one-step transition kernel parametrized by the birth probability $\theta_{0}$. Let F be the set of all Beta distributions. We choose the prior on $\theta_{0}$ to be a Beta distribution. Then, the conditions of Theorem 3 are satisfied with $\epsilon_{n}=O\left(\frac{1}{\sqrt{n}}\right)$ for any initial distribution $q^{\left(0\right)}$.*

Theorem 3 also applies to data generated by Markov chains with countably infinite state spaces, so long as the class of data-generating Markov chains is strongly ergodic and the initial distribution has finite second moments. The following example demonstrates this in the setting of a birth-death Markov chain on the positive integers, where the initial distribution is assumed to have finite second moments.

**Proposition** **9.** *Suppose the data-generating process is a birth-death Markov chain on the non-negative integers, parameterized by the probability of birth $\theta_{0}\in \left(0,\frac{1}{2}\right)$. Further let F be the set of all Beta distributions rescaled upon the support $\left(0,\frac{1}{2}\right)$. Let $q^{\left(0\right)}$ be a probability mass function on non-negative integers such that $\sum_{i=1}^{\infty }i^{2}q^{\left(0\right)}\left(i\right)<+\infty$. We choose the prior to be a scaled Beta distribution on (0,1/2) with parameters a and b. Then, the conditions of Theorem 3 are satisfied with $\epsilon_{n}=O\left(\frac{1}{\sqrt{n}}\right)$.*

Since continuous functions on a compact domain are bounded, we have the following (easy) corollary (stated without proof).

**Corollary** **2.** *Let $\left\{X_{0},\cdots ,X_{n}\right\}$ be generated by an α-mixing Markov chain parametrized by $\theta_{0}\in \Theta$ on a compact state space, and with initial distribution $q^{\left(0\right)}$. Suppose the α-mixing coefficients satisfy $\sum_{k\geq 1}\alpha_{k}^{\delta /\left(2+\delta \right)}<+\infty$, and that Assumption 1 holds with continuous functions $M_{k}^{\left(1\right)}\left(\cdot ,\cdot \right)$, k∈{1,2,⋯,m}. Furthermore, assume that there exists $\rho_{n}$ such that $K\left(\rho_{n},\pi \right)\leq \sqrt{n}C$ for some constant C. Then, Theorem 3 is satisfied with $\epsilon_{n}=O\left(\max(\frac{1}{\sqrt{n}},\frac{n^{\delta /2}}{n})\right)$.*

In general, the $M_{k}^{\left(1\right)}$ functions will not be uniformly bounded (consider the case of the Gauss–Markov simple linear model in Example 2), and stronger conditions must be imposed on the data-generating Markov chain itself. The following assumption imposes a ‘drift’ condition from [21]. Specifically, [21] (Theorem 2.3) shows that under the conditions of Assumption 2, the moment generating function of an aperiodic Markov chain $\left\{X_{n}\right\}$ can be upper bounded by a function of the moment generating function of $X_{0}$. Together with the α-mixing condition, Assumption 2 implies that this Markov data generating process satisfies Corollary 1.

**Assumption** **2.** *Consider a Markov chain $\left\{X_{n}\right\}$ parameterized by $\theta_{0}\in \Theta$. Let $M_{-\infty }^{n}$ denote the σ-field generated by $\left\{X_{-\infty },\cdots ,X_{n-1},X_{n}\right\}$. Denote the stochastic process $\left\{M_{n}^{k}\right\}:=\left\{M_{k}^{\left(1\right)}\left(X_{n},X_{n-1}\right)\right\}$; recall $M_{k}^{\left(1\right)}$, for each $k=1,\ldots ,m_{1}$, are defined in Assumption 1. For each k=1,…,m, assume the process $\left\{M_{n}^{k}\right\}$ satisfies the following conditions:*

- *The drift condition holds for $\left\{M_{n}^{k}\right\}$, i.e., $E\left[M_{n}^{k}-M_{n-1}^{k}|M_{-\infty }^{n-1},M_{n-1}^{k}>a\right]\leq -\epsilon$ for some ϵ,a>0.*
- *For some λ>0 and D>0, $E\left[e^{\lambda (M_{n}^{k}-M_{n-1}^{k})}|M_{-\infty }^{n-1}\right]\leq D$.*

Under this drift condition, the next theorem shows that Corollary 1 is satisfied.

**Theorem** **4.** *Let $\left\{X_{0},\ldots ,X_{n}\right\}$ be generated by an aperiodic α-mixing Markov chain parametrized by $\theta_{0}\in \Theta$ and initial distribution $q^{\left(0\right)}$. Suppose that Assumption 1 and Assumption 2 hold, and that the α-mixing coefficients satisfy $\sum_{k\geq 1}\alpha_{k}^{\delta /(2+\delta )}<+\infty$. Furthermore, assume $K\left(\rho_{n},\pi \right)\leq \sqrt{n}C$ for some constant C>0. If $\int e^{\lambda M_{k}^{\left(1\right)}\left(y,x\right)}p_{\theta_{0}}\left(y|x\right)q_{1}^{\left(0\right)}\left(x\right)dx<+\infty$ for all $k=1,\ldots ,m_{1}$, then the conditions of Corollary 1 are satisfied with $\epsilon_{n}=O\left(\max(\frac{1}{\sqrt{n}},\frac{n^{\delta /2}}{n})\right)$.*

Verifying the conditions in Theorem 4 can be quite challenging. Instead, we suggest a different approach that requires *f*-geometric ergodicity. Unlike the drift condition in Assumption 2, *f*-geometric ergodicity additionally requires the existence of a petite set. As noted before, geometric ergodicity implies α-mixing with geometrically decaying mixing coefficients. As with Theorem 4, we assume for simplicity that the Markov chain is aperiodic.

**Theorem** **5.** *Let $\left\{X_{0},\cdots ,X_{n}\right\}$ be generated by an aperiodic Markov chain parametrized by $\theta_{0}\in \Theta$ with known initial distribution $q^{\left(0\right)}$, and assumed to be V-geometrically ergodic for some $V:R^{m}\to \left[1,\infty \right)$. Suppose that Assumption 1 holds and $\int M_{k}^{\left(1\right)}{\left(y,x\right)}^{2+\delta }p_{\theta_{0}}\left(y|x\right)dy<V\left(x\right)\;\forall \;k,x\;\mathrm{and}\;\mathrm{some}\;\delta >0$. Furthermore, assume that $K\left(\rho_{n},\pi \right)\leq \sqrt{n}C$ for some constant C>0. If the initial distribution $q^{\left(0\right)}$ satisfies $E_{q^{\left(0\right)}}\left[V\left(X_{0}\right)\right]<+\infty$, then the conditions of Corollary 1 are satisfied with $\epsilon_{n}=O\left(\max(\frac{1}{\sqrt{n}},\frac{n^{\delta /2}}{n})\right)$.*

The following Proposition 10 shows, the simple linear model satisfies Theorem 5 when the parameter $\theta_{0}$ is suitably restricted.

**Proposition** **10.** *Consider the simple linear model satisfying the equation*

(22) $$\begin{array}{cc} X_{n} & =\theta_{0}X_{n-1}+W_{n}, \end{array}$$

*where $\left\{W_{n}\right\}$ are i.i.d. standard Gaussian random variables and $|\theta_{0}|<2^{\frac{1}{4+2\delta }-1}$ for δ>0. Let F be the space of all scaled Beta distributions on (−1,1) and suppose the prior π is a uniform distribution on (−1,1). Then, the conditions of Theorem 5 are satisfied with $\epsilon_{n}=O\left(\max(\frac{1}{\sqrt{n}},\frac{n^{\delta /2}}{n})\right)$, if the initial distribution $q^{\left(0\right)}$ satisfies $E_{q^{\left(0\right)}}\left[X_{0}^{4+2\delta }\right]<+\infty$.*

## 5. Misspecified Models

We show next how our results can be extended to the misspecified model setting. Assume that the true data generating distribution is parametrized by $\theta_{0}\notin \Theta$. Let $\theta_{n}^{*}:=\arg\min_{\theta \in \Theta }K\left(P_{\theta_{0}}^{\left(n\right)},P_{\theta }^{\left(n\right)}\right)$ represent the closest parametrized distribution in the variational family to the data-generating distribution. Further, assume our usual conditions:

*i.*  $\int E\left[r_{n}\left(\theta ,\theta_{n}^{*}\right)\right]\rho_{n}\left(d\theta \right)\leq n\epsilon_{n}$,
*ii.*  $\int \mathrm{Var}\left(r_{n}\left(\theta ,\theta_{n}^{*}\right)\right)\rho_{n}\left(d\theta \right)\leq n\epsilon_{n}$.

Now, since $r_{n}\left(\theta ,\theta_{0}\right)=r_{n}\left(\theta ,\theta_{n}^{*}\right)+r_{n}\left(\theta_{n}^{*},\theta_{0}\right)$, we have

(23) $$\;\int K\left(P_{\theta_{0}}^{\left(n\right)},P_{\theta }^{\left(n\right)}\right)\rho_{n}\left(d\theta \right)\leq E\left[r_{n}\left(\theta_{0},\theta_{n}^{*}\right)\right]+n\epsilon_{n}.$$

Similarly, decomposing the variance it follows that

(24) $$\begin{array}{cc} \mathrm{Var}[r_{n}\left(\theta ,\theta_{0}\right)] & =\mathrm{Var}\left[r_{n}\left(\theta ,\theta_{n}^{*}\right)\right]+\mathrm{Var}\left[r_{n}\left(\theta_{n}^{*},\theta_{0}\right)\right]+2\mathrm{Cov}\left[r_{n}\left(\theta ,\theta_{n}^{*}\right),r_{n}\left(\theta_{n}^{*},\theta_{0}\right)\right]. \end{array}$$

Using the fact that $2ab\leq a^{2}+b^{2}$ on the covariance term $2\mathrm{Cov}\left[r_{n}\left(\theta ,\theta_{n}^{*}\right),r_{n}\left(\theta_{n}^{*},\theta_{0}\right)\right]=2E\left[\left(r_{n}\left(\theta ,\theta_{n}^{*}\right)-E\left[r_{n}\left(\theta ,\theta_{n}^{*}\right)\right]\right)\left(r_{n}\left(\theta_{n}^{*},\theta_{0}\right)-E\left[r_{n}\left(\theta_{n}^{*},\theta_{0}\right)\right]\right)\right]$, we have

(25) $$\begin{array}{cc} \mathrm{Var}[r_{n}\left(\theta ,\theta_{0}\right)] & \leq 2\mathrm{Var}\left[r_{n}\left(\theta ,\theta_{n}^{*}\right)\right]+2\mathrm{Var}\left[r_{n}\left(\theta_{n}^{*},\theta_{0}\right)\right]. \end{array}$$

Integrating both sides with respect to $\rho_{n}\left(d\theta \right)$ we get

(26) $$\begin{array}{cc} \int \mathrm{Var}\left[r_{n}\left(\theta ,\theta_{0}\right)\right]\rho_{n}\left(d\theta \right) & \leq 2\int \mathrm{Var}\left[r_{n}\left(\theta ,\theta_{n}^{*}\right)\right]\rho_{n}\left(d\theta \right)+2\int \mathrm{Var}\left[r_{n}\left(\theta_{n}^{*},\theta_{0}\right)\right]\rho_{n}\left(d\theta \right)\\ \; & \leq 2n\epsilon_{n}+2\mathrm{Var}\left[r_{n}\left(\theta_{n}^{*},\theta_{0}\right)\right].\; \end{array}$$

Consequently, we arrive at the following result:

**Theorem** **6.** Let F be a subset of all probability distributions parameterized by *Θ*. Let $\theta_{n}^{*}=\arg\min_{\theta \in \Theta }K\left(P_{\theta_{0}}^{\left(n\right)},P_{\theta }^{\left(n\right)}\right)$ and assume there exist $\epsilon_{n}>0$ and $\rho_{n}\in F$ such that

*i*.  $\int E\left[r_{n}\left(\theta ,\theta_{n}^{*}\right)\right]\rho_{n}\left(d\theta \right)\leq n\epsilon_{n}$,
*ii*.  $\int \mathrm{Var}\left(r_{n}\left(\theta ,\theta_{n}^{*}\right)\right)\rho_{n}\left(d\theta \right)\leq n\epsilon_{n}$, and
*iii*.  *$K\left(\rho_{n},\pi \right)\leq n\epsilon_{n}$.*

*Then, for any $\alpha^{re}\in \left(0,1\right)$ and (ϵ,η)∈(0,1)×(0,1),*

(27) $$\begin{array}{cc} \;P[\int D_{\alpha^{re}} & \left(P_{\theta }^{\left(n\right)},P_{\theta_{0}}^{\left(n\right)}\right){\tilde{\pi }}_{n,\alpha^{re}}\left(d\theta |X^{\left(n\right)}\right)\leq \\ \; & \frac{\left(\alpha^{re}+1\right)n\epsilon_{n}+E\left[r_{n}\left(\theta_{0},\theta_{n}^{*}\right)\right]+\alpha^{re}\sqrt{\frac{2n\epsilon_{n}+2\mathrm{Var}\left[r_{n}\left(\theta_{n}^{*},\theta_{0}\right)\right]}{\eta }}-\log\left(\epsilon \right)}{1-\alpha^{re}}]\geq 1-\epsilon -\eta . \end{array}$$

The proof of this theorem is straightforward and follows from the proof of Theorem 1 by plugging in the upper bounds for KL-divergence from Equation (23), and variance from Equation (26) to (A13). A sketch of the proof is presented in the appendix.

## 6. Conclusions

Concentration of the KL-VB model risk, in terms of the expected $\alpha^{re}$-Rényi divergence, is well established under the i.i.d. data generating model assumption. Here, we extended this to the setting of Markov data generating models, linking the concentration rate to the mixing and ergodic properties of the Markov model. Our results apply to both stationary and non-stationary Markov chains, as well as to the situation with misspecified models. There remain a number of open questions. An immediate one is to extend the current analysis to continuous-time Markov chains and Markov jump processes, possibly using uniformization of the continuous time model. Another direction is to extend this to the setting of non-homogeneous Markov chains, where analogues of notions such as stationarity are less straightforward. Further, as noted in the introduction, [14] establish PAC-Bayes bounds under slightly weaker ‘existence of test functions’ conditions, while our results are established under the stronger conditions used by [15] for the i.i.d. setting. Weakening the conditions in our analysis is important, but complicated. A possible path is to build on results from [22], who provides conditions form the existence of exponentially powerful test functions exist for distinguishing between two Markov chains. It is also known that there exists a likelihood ratio test separating any two ergodic measures [23]. However, leveraging these to establish the PAC-Bayes bounds for the KL-VB posterior is a challenging effort that we leave to future papers. Finally it is of interest to generalize our PAC-bounds to posterior approximations beyond KL-variational inference, such as $\alpha^{re}$-Rényi posterior approximations [6], and loss-calibrated posterior approximations [24,25].

## Appendix A. Technical Desiderata

### Appendix A.1. Definitions Related to Markov Chains

As noted before, ergodicity plays an acute role in establishing our results. We consolidate various definitions used throughout the paper in this appendix. Recall that we assume the parameterized Markov chain possesses an invariant probability density or mass function $q_{\theta }$ under parameter θ∈Θ. Our results in Section 4 also rely on the ergodic properties of the Markov chain, and we assume that the Markov chain is *f*-geometrically ergodic [20] (Chapter 15). First, refer to the definition of the functional norm ${∥\cdot ∥}_{f}$, from Definition A.1.1,

**Definition** **A.1.1** (*f*-norm)**.** *The functional norm in f-metric of a measure v, or the f-norm of v is*

(A1) $${∥v∥}_{f}=\underset{g:|g|<f}{\sup}\left|\int gdv\right|,$$

*where f and g are any two functions.*

An immediate consequence of this definition is that if $f_{1},f_{2}$ are two functions such that $f_{1}<f_{2}$ (i.e., for all points in the support of the functions), then

(A2) $${\;∥v∥}_{f_{1}}\leq {∥v∥}_{f_{2}}.$$

Now that we have defined the ${∥\cdot ∥}_{f}$ norm, we can now define *f*-geometric ergodicity. In the following, we assume the Markov chain is positive Harris; see [20] for a definition. This is a mild and fairly standard assumption in Markov chain theory.

**Definition** **A.1.2** (*f*-geometric ergodicity)**.** *For any function f, Markov chain $\left\{X_{n}\right\}$ parameterized by θ∈Θ is said to be f-geometrically ergodic if it is positive Harris and there exists a constant $r_{f}>1$, that depends on f, such that for any A∈B(X),*

(A3) $$\sum_{n=1}^{n}r_{f}^{n}{∥P_{\theta }\left(X_{n}\in A|X_{0}=x\right)-\int_{A}q_{\theta }\left(y\right)dy∥}_{f}<\infty .$$

It is straightforward to see that this is equivalent to

$${∥P_{\theta }\left(X_{n}\in A|X_{0}=x\right)-\int q_{\theta }\left(y\right)dy∥}_{f}\leq Cr_{f}^{-n}$$

for an appropriate constant *C* (which may depend on the state *x*), that is, the Markov chain approaches steady state at a geometrically fast rate. If a Markov chain is *f*-geometrically ergodic for f≡1, then, it is simply termed as *geometrically ergodic*. It is straightforward to see (via Theorem A.1.2 in the Appendix) that a geometrically ergodic Markov chain is also α-mixing, with mixing coefficients satisfying

(A4) $$\sum_{k\geq 0}\alpha_{k}^{\upsilon }<\infty \;\forall \;\upsilon >0,$$

showing that, under geometric ergodicity, the α-mixing coefficients raised to any positive power υ are finitely summable. We note here that the most standard procedure to establish *f*-geometric ergodicity for any Markov chain is through the verification of the drift condition. The drift condition is a sufficient condition for a Markov chain to be *f*-geometrically ergodic, as long as there exists a set (called petite set) towards which the Markov chain drifts to (see Assumption A.1.1 in the appendix). If a Markov chain is *f*-geometrically ergodic with f≡V, for some particular function *V*, then we call it *V*-geometrically ergodic.

We defined *V*-geometric ergodicity in the previous sections. In this section, we provide a sufficient condition for a Markov chain to be *V*-geometrically ergodic. First, we recall the definition of resolvent from [20] (Chapter 5).

**Definition** **A.1.3** (Resolvent)**.** *Let n∈{0,1,2,⋯} and $q_{n}$ be such that $q_{n}\geq 0\;\forall \;n$ and $\sum_{n=1}^{\infty }q_{n}=1$. Note that $q_{n}$ can be thought of being a probability mass function for a random variable "q" taking values on non-negative integers. Then, the resolvent of a Markov chain with respect to q is given by $K_{q}\left(x,A\right)$ where,*

(A5) $$\begin{array}{cc} K_{q}\left(x,A\right) & =\sum_{n=0}^{\infty }q_{n}P\left(X_{n}\in A|X_{0}=x\right). \end{array}$$

Then, the definition of petite sets follows (see, for Reference, [20] (Chapter 5)).

**Definition** **A.1.4** (Petite Sets)**.** *Let $X_{0},\cdots ,X_{n}$ be n samples from a Markov chain taking values on the state space X. Let C be a set. We shall call C to be $v_{q}$ petite if*

$$\begin{array}{c} K_{q}\left(x,B\right)\geq \upsilon_{q}\left(B\right) \end{array}$$

*for all x∈C and B∈B(X), and a non-trivial measure $\upsilon_{q}$ on B(X), and a probability mass function q on {1,2,3,⋯}*

Now, let $\Delta V\left(x\right):=E\left[V\left(X_{n}\right)|X_{n-1}=x\right]-V\left(x\right)$ for V:S→[1,∞).

**Assumption** **A.1.1** (Drift condition)**.** *[20] (Chapter 5) Suppose the chain $\left\{X_{n}\right\}$ is, aperiodic and ψ-irreducible. Let there exists a petite set C, constants b<∞,β>0, and a non-trivial function V:S→[1,∞) satisfying*

(A6) $$\begin{array}{c} \Delta V\left(x\right)\leq -\beta V\left(x\right)+bI_{x\in C}\;\forall x\in S. \end{array}$$

If a Markov chain drifts towards a petite set then it is *V*-geometrically ergodic. Suppose, for simplicity, that V(x)=|X|. Then, the drift condition becomes $E[|X_{n}∥X_{x-1}]-|X_{n-1}\left|=-\beta \right|X_{n}|+bI_{X_{n}\in C}$. The left hand side of this equation represents the change in the state of the Markov chain in one time epoch. Thus, the condition in Assumption A.1.1 essentially states that the Markov chain drifts towards a petite set *C* and then, once it reaches that set, moves to any point in the state space with at least some probability independent of *C*.

**Theorem** **A.1.1** (Geometrically ergodic theorem)**.** *Suppose that $\left\{X_{n}\right\}$ is satisfies Assumption A.1.1. Then, the set $S_{V}=\left\{x:V\left(x\right)<\infty \right\}$ is absorbing, i.e., $P_{\theta }\left(X_{1}\in S_{V}|X_{0}=x\right)=1$$\forall x\in S_{V}$, and full, i.e., $\psi (S_{V}^{c})=0$. Furthermore, ∃constantsr>1,R<∞ such that, for any A∈B(S),*

(A7) $${∥P_{\theta }\left(X_{n}\in A|X_{0}=x\right)-\int_{A}q_{\theta }\left(y\right)dy∥}_{V}\leq Rr^{-n}V\left(x\right).$$

Any aperiodic and ψ-irreducible Markov chain satisfying the drift condition is geometrically ergodic. A consequence of Equation (A2) is that if, $\left\{X_{n}\right\}$ is *V*-geometrically ergodic, then for any other function U,suchthat|U|<V, it is also *U*-geometrically ergodic. In essence, a geometrically ergodic Markov chain is asymptotically uncorrelated in a precise sense. Recall ρ-mixing coefficients defined as follows. Let A be a sigma field and $L^{2}\left(A\right)$ be the set of square integrable, real valued, A measurable functions.

**Definition** **A.1.5** (ρ-mixing coefficient)**.** *Let $M_{i}^{j}$ denote the sigma field generated by the measures $X_{k},\;\mathrm{where}\;i\leq k\leq j$. Then,*

(A8) $$\rho_{k}=\underset{t>0}{\sup}\underset{\left(f,g\right)\in L^{2}\left(M_{-\infty }^{t}\right)\times L^{2}\left(M_{t+k}^{\infty }\right)}{\sup}\left|\mathrm{Corr}(f,g)\right|,$$

*where Corr is the correlation function.*

**Theorem** **A.1.2.**
*If $X_{n}$ is geometrically ergodic, then it is α-mixing. That is, there exists a constant c>0 such that $\alpha_{k}=O\left(e^{-ck}\right)$.*

**Proof.** By [26] (Theorem 2) it follows that a geometrically ergodic Markov chain is asymptotically uncorrelated with ρ-mixing coefficients (see Definition A.1.5) that satisfy $\rho_{k}=O\left(e^{-ck}\right)$. Furthermore, it is well known that [18,26] $\alpha_{k}\leq \frac{1}{4}\rho_{k}$, implying $\alpha_{k}=O\left(e^{-ck}\right)$. □

### Appendix A.2. Bounding the KL-Divergence between Beta Distributions

The following results will be utilized in the proofs of Propositions 8–10.

**Lemma** **A.2.1.**
*Let $\theta_{0}\in \left(0,1\right)$. Let, $\rho_{n}$ be a sequence of Beta distributions with parameters $a_{n}=n\theta_{0}$ and $b_{n}=n\left(1-\theta_{0}\right)$. Let π denote an uniform distribution, U(0,1). Then, $K\left(\rho_{n},\pi \right)<C+\frac{1}{2}\log\left(n\right)$, for some constant C>0.*

**Proof.** Without loss of generality, we can assume $a_{n}>1$ and $b_{n}>1$. The same form of the result can be obtained in all the other cases, by appropriate use of the bounds presented in the proof. We write the KL divergence $K(\rho_{n},\pi )$ as $\int \log\left(\frac{\rho_{n}}{\pi }\right)\rho_{n}\left(d\theta \right)$. Since π is uniform, π(θ)=1 whenever θ∈(0,1). Hence, the KL-divergence can be written as the negative of the entropy of $\rho_{n}$ $\int_{0}^{1}\log\left(\rho_{n}\left(\theta \right)\right)\rho_{n}\left(d\theta \right),$ which can be written as

(A9) $$\begin{array}{c} K\left(\rho_{n},\pi \right)=\left(a_{n}-1\right)\psi \left(a_{n}\right)+\left(b_{n}-1\right)\psi \left(b_{n}\right)-\left(a_{n}+b_{n}-2\right)\psi \left(a_{n}+b_{n}\right)\\ \;\;\;\;-\log\mathrm{Beta}(a_{n},b_{n}), \end{array}$$

where ψ is the digamma function. Using Stirling’s approximation on $\mathrm{Beta}(a_{n},b_{n})$ yields,

$$\begin{array}{c} \mathrm{Beta}\left(a_{n},b_{n}\right)=\sqrt{2\pi }\frac{a_{n}^{a_{n}-1/2}b_{n}^{b_{n}-1/2}}{{\left(a_{n}+b_{n}\right)}^{a_{n}+b_{n}-1/2}}\left(1+o\left(1\right)\right). \end{array}$$

Hence, setting $C_{1}=\log\left(2\sqrt{\pi }\right)$, we can write $-\log\mathrm{Beta}(a_{n},b_{n})$ as,

$$\begin{array}{cc} -\log\mathrm{Beta}(a_{n},b_{n}) & =C_{1}-\left(a_{n}-\frac{1}{2}\right)\log\left(a_{n}\right)-\left(b_{n}-\frac{1}{2}\right)\log\left(b_{n}\right)\\ \; & \;+\left(a_{n}+b_{n}-\frac{1}{2}\right)\log\left(a_{n}+b_{n}\right)+\log\left(1+o\left(1\right)\right). \end{array}$$

From [27] we have that $\log\left(x\right)-\frac{1}{x}<\psi \left(x\right)<\log\left(x\right)-\frac{1}{2x}\;\forall \;x>0$. Since we assumed $a_{n}>1$ and $b_{n}>1$, the fact that $\psi \left(x\right)<\log\left(x\right)-\frac{1}{2x}$ implies

$$\begin{array}{cc} \left(a_{n}-1\right)\psi \left(a_{n}\right) & <\left(a_{n}-1\right)\log\left(a_{n}\right)-\frac{a_{n}-1}{2a_{n}}\;\mathrm{and},\;\\ \left(b_{n}-1\right)\psi \left(b_{n}\right) & <\left(b_{n}-1\right)\log\left(b_{n}\right)-\frac{b_{n}-1}{2b_{n}}. \end{array}$$

Finally, using the fact that $\log\left(x\right)-\frac{1}{x}<\psi \left(x\right)$, we get,

$$\begin{array}{c} -\left(a_{n}+b_{n}-2\right)\psi \left(a_{n}+b_{n}\right)<-\left(a_{n}+b_{n}-2\right)\log\left(a_{n}+b_{n}\right)+\frac{a_{n}+b_{n}-2}{a_{n}+b_{n}}. \end{array}$$

Therefore, after much cancellation, the KL-divergence

$$\begin{array}{c} \left(a_{n}-1\right)\psi \left(a_{n}\right)+\left(b_{n}-1\right)\psi \left(b_{n}\right)-\left(a_{n}+b_{n}-2\right)\psi \left(a_{n}+b_{n}\right)-\log\mathrm{Beta}\left(a_{n},b_{n}\right) \end{array}$$

can be upper bounded by

$$\begin{array}{c} -\frac{1}{2}\log\left(a_{n}\right)-\frac{1}{2}\log\left(b_{n}\right)+\frac{3}{2}\log\left(a_{n}+b_{n}\right)+\frac{a_{n}+b_{n}-2}{a_{n}+b_{n}}-\frac{a_{n}-1}{2a_{n}}-\frac{b_{n}-1}{2b_{n}}. \end{array}$$

Now, plugging in the values of $a_{n}$ and $b_{n}$, we get Plugging in the values of $a_{n}$ and $b_{n}$, we get as upper bound for the KL-divergence as,

$$\begin{array}{cc} K(\rho_{n},\pi ) & <-\frac{1}{2}\log\left(n\theta_{0}\right)-\frac{1}{2}\log\left(n\left(1-\theta_{0}\right)\right)+\frac{3}{2}\log\left(n\right)+\frac{n-2}{n}-\frac{n\theta_{0}-1}{2n\theta_{0}}-\frac{n(1-\theta_{0})-1}{2n(1-\theta_{0})}\\ \; & =\frac{1}{2}\log\left(n\right)-\frac{1}{2}\left(\log\left(\theta_{0}\right)+\log\left(1-\theta_{0}\right)\right)+3-\frac{2}{n}-\frac{1}{2n\theta_{0}}-\frac{1}{2n(1-\theta_{0})}\\ \; & <C+\frac{1}{2}\log\left(n\right), \end{array}$$

for some large enough positive constant *C*. This completes our proof. □

**Proposition** **A.2.1.**
*Let $\theta_{0}\in \left(0,1\right)$. Let, $\rho_{n}$ be a sequence of Beta distributions with parameters $a_{n}=n\theta_{0}$ and $b_{n}=n\left(1-\theta_{0}\right)$. Let π denote an Beta distribution, with parameters (a,b). Then, $K\left(\rho_{n},\pi \right)<C+\frac{1}{2}\log\left(n\right)$, for some constant C>0.*

**Proof.** Without loss of generality, we assume a>1 and b>1. As mentioned in the proof of Lemma A.2.1, the other cases follows similarly. We write the KL-divergence between $\rho_{n}$ and π as,

$$\begin{array}{cc} K(\rho_{n},\pi )= & \int \log\left(\frac{\rho_{n}}{\pi }\right)\rho_{n}\left(d\theta \right)=\int \log\left(\frac{\rho_{n}}{U}\right)\rho_{n}\left(d\theta \right)+\int \log\left(\frac{U}{\pi }\right)\rho_{n}\left(d\theta \right), \end{array}$$

where, *U* is an uniform distribution on (0,1). We analyze the second term in the above expression. The second term can be written as,

$$\begin{array}{cc} \int \log\left(\frac{U}{\pi }\right)\rho_{n}\left(d\theta \right) & =\int \log\left(\frac{1}{\frac{1}{\mathrm{Beta}(a,b)}\theta^{a-1}{\left(1-\theta \right)}^{b-1}}\right)\rho_{n}\left(d\theta \right)\\ \; & =C_{1}-\left(a-1\right)\int \log\left(\theta \right)\rho_{n}\left(d\theta \right)-\left(b-1\right)\int \log\left(1-\theta \right)\rho_{n}\left(d\theta \right), \end{array}$$

where $C_{1}$ is log(Beta(a,b)). Since, $\rho_{n}$ follows a Beta distribution with parameters $a_{n}=n\theta_{0}$ and $b_{n}=n\left(1-\theta_{0}\right)$, we get that,

$$\begin{array}{cc} \int \log\left(\frac{U}{\pi }\right)\rho_{n}\left(d\theta \right) & =C_{1}-\left(a-1\right)\left[\psi \left(a_{n}\right)-\psi \left(a_{n}+b_{n}\right)\right]-\left(b-1\right)\left[\psi \left(b_{n}\right)-\psi \left(a_{n}+b_{n}\right)\right] \end{array}$$

Since, $\log\left(x\right)-\frac{1}{x}<\psi \left(x\right)<\log\left(x\right)-\frac{1}{2x}$, looking at the term $\left[\psi \left(a_{n}\right)-\psi \left(a_{n}+b_{n}\right)\right]$, we get that,

$$\begin{array}{cc} -\left[\psi \left(a_{n}\right)-\psi \left(a_{n}+b_{n}\right)\right] & =-\left[\psi \left(n\theta_{0}\right)-\psi \left(n\theta_{0}+n\left(1-\theta_{0}\right)\right)\right]\\ \; & =-\left[\psi \left(n\theta_{0}\right)-\psi \left(n\right)\right]. \end{array}$$

Using the lower bound on $\psi (n\theta_{0})$ and the upper bound on ψ(n), we get

$$\begin{array}{cc} -\left[\psi \left(a_{n}\right)-\psi \left(a_{n}+b_{n}\right)\right] & <-\log\left(n\theta_{0}\right)+\frac{1}{n\theta_{0}}+\log\left(n\right)-\frac{1}{2n}\\ & =-\log\left(\theta_{0}\right)+\frac{2-\theta_{0}}{2n\theta_{0}}. \end{array}$$

Furthermore, similarly, we get that,

$$\begin{array}{cc} -\left[\psi \left(b_{n}\right)-\psi \left(a_{n}+b_{n}\right)\right] & <-\log\left(1-\theta_{0}\right)+\frac{2-(1-\theta_{0})}{2n(1-\theta_{0})}. \end{array}$$

Therefore it follows that

$$\begin{array}{cc} \max & \left\{-\left(a-1\right)\left[\psi \left(a_{n}\right)-\psi \left(a_{n}+b_{n}\right)\right],-\left(b-1\right)\left[\psi \left(b_{n}\right)-\psi \left(a_{n}+b_{n}\right)\right]\right\}\\ \; & <\max\left\{\left(a-1\right)\left[-\log\left(\theta_{0}\right)+\frac{2-\theta_{0}}{2n\theta_{0}}\right],\left(b-1\right)\left[-\log\left(1-\theta_{0}\right)+\frac{2-(1-\theta_{0})}{2n(1-\theta_{0})}\right]\right\}\\ \; & <C, \end{array}$$

for a large positive constant *C*. Using the above bounds, we finally show that,

$$\begin{array}{c} C_{1}-\left(a-1\right)\left[\psi \left(a_{n}\right)-\psi \left(a_{n}+b_{n}\right)\right]-\left(b-1\right)\left[\psi \left(b_{n}\right)-\psi \left(a_{n}+b_{n}\right)\right]\\ \;\;\;\;\;<C_{1}+2C, \end{array}$$

which can be upper bounded by $C^{\prime }$ for some large constant $C^{\prime }$. Finally, we upper bound $\int \log\left(\frac{\rho_{n}}{U}\right)\rho_{n}\left(d\theta \right)$ by Lemma A.2.1 thereby completing the proof. □

## Appendix B. Proofs of Main Results

### Appendix B.1. Proofs for A Concentration Bound for the α^re^-Rényi Divergence

#### Appendix B.1.1. Proof of Proposition 1

We start by recalling the variational formula of Donsker and Varadhan [28].

**Lemma** **B.1.1** (Donsker-Varadhan)b**.** *For any probability distribution function π on Θ, and for any measurable function h:Θ→R, if $\int e^{h}d\pi <\infty$, then*

(A10) $$\begin{array}{cc} \log\int e^{h}d\pi & =\underset{\rho \in M^{+}\left(\Theta \right)}{\sup}\left\{\int hd\rho -K(\rho ,\pi )\right\} \end{array}$$

Now, fix $\alpha^{re}\in \left(0,1\right)$, and θ∈Θ. First, observe that by the definition of the $\alpha^{re}$-Rényi divergence we have

$$\begin{array}{cc} \; & E_{\theta_{0}}^{\left(n\right)}\left[\exp\left(-\alpha^{re}r_{n}\left(\theta ,\theta_{0}\right)\right)\right]=\exp\left[-\left(1-\alpha^{re}\right)D_{\alpha^{re}}\left(P_{\theta }^{\left(n\right)},P_{\theta_{0}}^{\left(n\right)}\right)\right] \end{array}$$

Multiplying both sides of the equation by $\exp[\left(1-\alpha^{re}\right)D_{\alpha^{re}}\left(P_{\theta }^{\left(n\right)},P_{\theta_{0}}^{\left(n\right)}\right)$ and integrating with respect to (w.r.t.) π(θ) it follows that

$$\begin{array}{c} \int E_{\theta_{0}}^{\left(n\right)}\left[\exp\left(-\alpha^{re}r_{n}\left(\theta ,\theta_{0}\right)+\left(1-\alpha^{re}\right)D_{\alpha^{re}}\left(P_{\theta }^{\left(n\right)},P_{\theta_{0}}^{\left(n\right)}\right)\right)\right]\pi \left(d\theta \right)=1,\mathrm{or} \end{array}$$

$$\begin{array}{cc} \;\; & E_{\theta_{0}}^{\left(n\right)}\left[\int \exp\left(-\alpha^{re}r_{n}\left(\theta ,\theta_{0}\right)+\left(1-\alpha^{re}\right)D_{\alpha^{re}}\left(P_{\theta }^{\left(n\right)},P_{\theta_{0}}^{\left(n\right)}\right)\right)\pi \left(d\theta \right)\right]=1. \end{array}$$

Define $h\left(\theta \right):=-\alpha^{re}r_{n}\left(\theta ,\theta_{0}\right)+\left(1-\alpha^{re}\right)D_{\alpha^{re}}\left(P_{\theta }^{\left(n\right)},P_{\theta_{0}}^{\left(n\right)}\right)$. Then, applying Lemma B.1.1 to the integrand on the left hand side (l.h.s.) above, it follows that

$$\begin{array}{c} E_{\theta_{0}}^{\left(n\right)}\left[\exp\left(\underset{\rho \in M^{+}\left(\Theta \right)}{\sup}\left[\int h(\theta )\rho (d\theta )-K(\rho ,\pi )\right]\right)\right]=1. \end{array}$$

Multiply both sides of this equation by ϵ>0 to obtain

$$\begin{array}{c} E_{\theta_{0}}^{\left(n\right)}\left[\exp\left(\underset{\rho \in M^{+}\left(\Theta \right)}{\sup}\left[\int h(\theta )\rho (d\theta )-K(\rho ,\pi )+\log(\epsilon )\right]\right)\right]=\epsilon . \end{array}$$

Now, by Markov’s inequality, we have

(A11) $$P_{\theta_{0}}^{\left(n\right)}\left[\underset{\rho \in M^{+}\left(\Theta \right)}{\sup}\int \left(-\alpha^{re}r_{n}\left(\theta ,\theta_{0}\right)+\left(1-\alpha^{re}\right)D_{\alpha^{re}}\left(P_{\theta }^{\left(n\right)},P_{\theta_{0}}^{\left(n\right)}\right)\right)\rho \left(d\theta \right)-K\left(\rho ,\pi \right)+\log\left(\epsilon \right)\geq 0\right]\leq \epsilon .$$

Thus, it follows via complementation that

$$\begin{array}{cc} P_{\theta_{0}}^{\left(n\right)}[\forall \rho \in F\left(\Theta \right)\int D_{\alpha^{re}}\left(P_{\theta }^{\left(n\right)},P_{\theta_{0}}^{\left(n\right)}\right)\rho \left(d\theta \right)\leq \frac{\alpha^{re}}{\left(1-\alpha^{re}\right)}\int r_{n}\left(\theta ,\theta_{0}\right)\rho \left(d\theta \right)+ & \frac{K(\rho ,\pi )-\log(\epsilon )}{1-\alpha^{re}}]\\ \; & \geq 1-\epsilon , \end{array}$$

thereby completing the proof.

#### Appendix B.1.2. Proof of Theorem 1

Recall the definition of the fractional posterior and the VB approximation,

$$\begin{array}{c} \pi_{n,\alpha^{re}|X^{n}}=\frac{\exp^{-\alpha^{re}r_{n}\left(\theta ,\theta_{0}\right)\left(X^{n}\right)}\pi \left(d\theta \right)}{\int \exp^{-\alpha^{re}r_{n}\left(\gamma ,\theta_{0}\right)\left(X^{n}\right)}\pi \left(d\gamma \right)},\;{\tilde{\pi }}_{n,\alpha^{re}|X^{n}}=\underset{\rho \in F}{\arg\min}\;K\left(\rho ,\pi_{n,\alpha^{re}|X^{\left(n\right)}}\right). \end{array}$$

It follows by definition of the KL divergence that

(A12) $$\begin{array}{cc} {\tilde{\pi }}_{n,\alpha^{re}|X^{n}} & =\underset{\rho \in F}{\arg\min}\left\{-\alpha^{re}\int r_{n}\left(\theta ,\theta_{0}\right)\rho \left(d\theta \right)+K\left(\rho ,\pi \right)\right\}, \end{array}$$

where π is the prior distribution. Following Proposition 1 it follows that for any ϵ>0

$$\int D_{\alpha^{re}}\left(P_{\theta }^{\left(n\right)},P_{\theta_{0}}^{\left(n\right)}\right)\tilde{\pi }\left(d\theta |X^{n}\right)\leq \frac{\alpha^{re}}{\left(1-\alpha^{re}\right)}\int r_{n}\left(\theta ,\theta_{0}\right)\rho \left(d\theta \right)+\frac{K(\rho ,\pi )-\log(\epsilon )}{1-\alpha^{re}},$$

with probability 1−ϵ. We fix an η∈(0,1). Using Chebychev’s inequality, we have

$$\begin{array}{cc} \; & P_{\theta_{0}}^{\left(n\right)}[\frac{\alpha^{re}}{1-\alpha^{re}}\int r_{n}\left(\theta ,\theta_{0}\right)\rho_{n}\left(d\theta \right)\geq \frac{\alpha^{re}}{1-\alpha^{re}}\int E\left[r_{n}\left(\theta ,\theta_{0}\right)\right]\rho_{n}\left(d\theta \right)\\ \; & \;\;+\frac{\alpha^{re}}{1-\alpha^{re}}\sqrt{\frac{\mathrm{Var}[\int r_{n}\left(\theta ,\theta_{0}\right)\rho_{n}\left(d\theta \right)]}{\eta }}+\frac{K(\rho_{n},\pi )}{1-\alpha^{re}}]\\ \; & =P_{\theta_{0}}^{\left(n\right)}[\frac{\alpha^{re}}{1-\alpha^{re}}\int r_{n}\left(\theta ,\theta_{0}\right)\rho_{n}\left(d\theta \right)-\frac{\alpha^{re}}{1-\alpha^{re}}\int E\left[r_{n}\left(\theta ,\theta_{0}\right)\right]\rho_{n}\left(d\theta \right)-\frac{K(\rho_{n},\pi )}{1-\alpha^{re}}\\ \; & \;\;\geq \frac{\alpha^{re}}{1-\alpha^{re}}\sqrt{\frac{\mathrm{Var}[\int r_{n}\left(\theta ,\theta_{0}\right)\rho_{n}\left(d\theta \right)]}{\eta }}]\\ \; & \leq \frac{\mathrm{Var}\left[\frac{\alpha^{re}}{1-\alpha^{re}}\int r_{n}\left(\theta ,\theta_{0}\right)\rho_{n}\left(d\theta \right)-\frac{\alpha^{re}}{1-\alpha^{re}}\int E\left[r_{n}\left(\theta ,\theta_{0}\right)\right]\rho_{n}\left(d\theta \right)-\frac{K(\rho_{n},\pi )}{1-\alpha^{re}}\right]}{\frac{{\left(\alpha^{re}\right)}^{2}}{{\left(1-\alpha^{re}\right)}^{2}}\frac{\mathrm{Var}[\int r_{n}\left(\theta ,\theta_{0}\right)\rho_{n}\left(d\theta \right)]}{\eta }}. \end{array}$$

Note that $\frac{\alpha^{re}}{1-\alpha^{re}}\int E\left(r_{n}\left(\theta ,\theta_{0}\right)\right)\rho_{n}\left(d\theta \right)$ and $\frac{K(\rho_{n},\pi )}{1-\alpha^{re}}$ are constants with respect to the data, implying

$$\begin{array}{cc} \mathrm{Var}[\frac{\alpha^{re}}{1-\alpha^{re}} & \int r_{n}\left(\theta ,\theta_{0}\right)\rho_{n}\left(d\theta \right)-\frac{\alpha^{re}}{1-\alpha^{re}}\int E\left[r_{n}\left(\theta ,\theta_{0}\right)\right]\rho_{n}\left(d\theta \right)-\frac{K(\rho_{n},\pi )}{1-\alpha^{re}}]\\ & \;=\frac{{\left(\alpha^{re}\right)}^{2}}{{\left(1-\alpha^{re}\right)}^{2}}\mathrm{Var}\left[\int r_{n}\left(\theta ,\theta_{0}\right)\rho_{n}\left(d\theta \right)\right]. \end{array}$$

Therefore, we have

$$\begin{array}{cc} P_{\theta_{0}}^{\left(n\right)}[\frac{\alpha^{re}}{1-\alpha^{re}}\int r_{n}\left(\theta ,\theta_{0}\right)\rho_{n}\left(d\theta \right)\geq \frac{\alpha^{re}}{1-\alpha^{re}} & \int E\left[r_{n}\left(\theta ,\theta_{0}\right)\right]\rho_{n}\left(d\theta \right)\\ & +\frac{\alpha^{re}}{1-\alpha^{re}}\sqrt{\frac{\mathrm{Var}[\int r_{n}\left(\theta ,\theta_{0}\right)\rho_{n}\left(d\theta \right)]}{\eta }}+\frac{K(\rho_{n},\pi )}{1-\alpha }]\leq \eta . \end{array}$$

From Proposition 1, with probability 1−ϵ the following holds

$$\int D_{\alpha^{re}}\left(P_{\theta }^{\left(n\right)},P_{\theta_{0}}^{\left(n\right)}\right){\tilde{\pi }}_{n,\alpha^{re}|X^{n}}\left(d\theta \right)\leq \frac{\alpha^{re}\int r_{n}\left(\theta ,\theta_{0}\right)\rho_{n}\left(d\theta \right)+K\left(\rho_{n},\pi \right)-\log\left(\epsilon \right)}{1-\alpha^{re}}.$$

Therefore, with probability 1−η−ϵ the following statement holds

(A13) $$\begin{array}{cc} \;\int D_{\alpha^{re}}\left(P_{\theta }^{\left(n\right)},P_{\theta_{0}}^{\left(n\right)}\right){\tilde{\pi }}_{n,\alpha^{re}|X^{n}}\left(d\theta \right) & \leq \frac{\alpha^{re}}{1-\alpha^{re}}\int K\left(P_{\theta_{0}}^{\left(n\right)},P_{\theta }^{\left(n\right)}\right)\rho_{n}\left(d\theta \right)\\ & \;+\frac{\alpha^{re}}{1-\alpha^{re}}\sqrt{\frac{\mathrm{Var}[\int r_{n}\left(\theta ,\theta_{0}\right)\rho_{n}\left(d\theta \right)]}{\eta }}\\ & \;+\frac{K\left(\rho_{n},\pi \right)-\log\left(\epsilon \right)}{1-\alpha^{re}}. \end{array}$$

Next, we observe that

$$\begin{array}{cc} \mathrm{Var}\left[\int r_{n}\left(\theta ,\theta_{0}\right)\rho_{n}\left(d\theta \right)\right] & =E_{\theta_{0}}^{\left(n\right)}\left[{\left|\int r_{n}\left(\theta ,\theta_{0}\right)\rho_{n}\left(d\theta \right)-E\left[\int r_{n}\left(\theta ,\theta_{0}\right)\rho_{n}\left(d\theta \right)\right]\right|}^{2}\right]\\ \; & \leq \int \mathrm{Var}\left[r_{n}\left(\theta ,\theta_{0}\right)\right]\rho_{n}\left(d\theta \right), \end{array}$$

by a straightforward application of Jensen’s inequality to the inner integral on the left hand side. Finally, following the hypotheses (i), (ii) and (iii), we have,

$$\begin{array}{cc} \int D_{\alpha^{re}}\left(P_{\theta }^{\left(n\right)},P_{\theta_{0}}^{\left(n\right)}\right){\tilde{\pi }}_{n,\alpha^{re}|X^{n}}\left(d\theta \right) & \leq \frac{\alpha^{re}}{1-\alpha^{re}}\int \left(K\left(P_{\theta_{0}}^{\left(n\right)},P_{\theta }^{\left(n\right)}\right)+\sqrt{\frac{\int \mathrm{Var}\left[r_{n}\left(\theta ,\theta_{0}\right)\right]\rho_{n}\left(d\theta \right)}{\eta }}\right)\rho_{n}\left(d\theta \right)\\ & \;+\frac{1}{\alpha^{re}}\left(K\left(\rho_{n},\pi \right)-\log\left(\epsilon \right)\right)\\ \; & \leq \frac{\alpha^{re}\left(\epsilon_{n}+\sqrt{\frac{n\epsilon_{n}}{\eta }}\right)}{1-\alpha^{re}}+\frac{n\epsilon_{n}-\log\left(\epsilon \right)}{1-\alpha^{re}}, \end{array}$$

thereby concluding the proof. □

#### Appendix B.1.3. Proof of Proposition 2

We define $Y_{i}:=\log\left(\frac{p_{\theta_{1}}\left(X_{i}|X_{i-1}\right)}{p_{\theta_{2}}\left(X_{i}|X_{i-1}\right)}\right)$ for i=1,…,n, and $Z_{0}=\log\left(\frac{q_{1}^{\left(0\right)}\left(X_{0}\right)}{q_{2}^{\left(0\right)}\left(X_{0}\right)}\right)$. Then, using the Markov property we can see that the Kullback–Leibler divergence between the joint distributions $P_{\theta_{1}}^{\left(n\right)}$ and $P_{\theta_{2}}^{\left(n\right)}$ satisfies $K\left(P_{\theta_{1}}^{\left(n\right)},P_{\theta_{2}}^{\left(n\right)}\right)=\sum_{i=1}^{n}E_{\theta_{1}}\left[Y_{i}\right]+E_{\theta_{1}}\left[Z_{0}\right].$ If the Markov chain $\left\{X_{i}\right\}$ is stationary under $\theta_{1}$, so is $\left\{Y_{i}\right\}$. Hence $Y_{i}\overset{d}{=}Y_{1}$ and the above equation reduces to,

(A14) $$\begin{array}{c} K\left(P_{\theta_{1}}^{\left(n\right)},P_{\theta_{2}}^{\left(n\right)}\right)=nE_{\theta_{1}}\left[Y_{1}\right]+E_{\theta_{1}}\left[Z_{0}\right]. \end{array}$$

□

#### Appendix B.1.4. Proof of Proposition 3

First, recall the following result from [19].

**Lemma** **B.1.2.**
*[19] (Lemma 1.2) Let $X_{-\infty },\cdots ,X_{1},X_{2},\cdots$ be an α-mixing Markov chain with α-mixing coefficients given by $\alpha_{k}$. Let $M_{a}^{b}$ be the sigma-field generated by the subsequence $\left(X_{a},X_{a+1},\cdots ,X_{b}\right)$. Let $\eta_{t}\in M_{-\infty }^{t}$ and $\tau_{t}\in M_{t+k}^{\infty }$ be adapted random variables such that $|\eta_{t}\left|\leq 1,\right|\tau_{t}|\leq 1$. Then,*

(A15) $$\begin{array}{c} \underset{t}{\sup}\;\underset{\eta_{t},\tau_{t}}{\sup}\left|E\left[\eta_{t}\tau_{t}\right]-E\left[\eta_{t}\right]E\left[\tau_{t}\right]\right|\leq 4\alpha_{k}. \end{array}$$

This lemma provides an upper bound on the covariance of events ηandτ, as shown next.

**Lemma** **B.1.3.**
*Let $\eta \in M_{-\infty }^{t}$$\tau \in M_{t+k}^{\infty }$ be such that, ${E|\eta |}^{2+\delta }\leq C_{1},E{\left|\tau \right|}^{2+\delta }\leq C_{2}\;\mathrm{for}\;\mathrm{some}\;\delta >0$. Then, for a fixed n<+∞, we have*

(A16) $$\left|E\eta \tau -E\eta E\tau \right|\leq \left(\frac{4}{n}+2n^{\delta /2}\left(C_{1}+C_{2}\right)+2n^{\delta /2}\sqrt{C_{1}C_{2}}\right)\alpha_{k}^{2\delta /(2+\delta )}.$$

**Proof.** Let N<+∞ be a fixed number. We get from the triangle inequality that

(A17) $$\begin{array}{cc} \left|E\eta \tau -E\eta E\tau \right| & \leq |E\eta \tau I_{\left[|\eta |\leq N,|\tau |\leq N\right]}-E\eta I_{\left[|\eta |\leq N\right]}E\tau I_{\left[|\tau |\leq N\right]}|\\ \; & \;+|E\eta \tau I_{\left[|\eta |\geq N,|\tau |\leq N\right]}-E\eta I_{\left[|\eta |\geq N\right]}E\tau I_{\left[|\tau |\leq N\right]}|\\ \; & \;+|E\eta \tau I_{\left[|\eta |\leq N,|\tau |\geq N\right]}-E\eta I_{\left[|\eta |\leq N\right]}E\tau I_{\left[|\tau |\geq N\right]}|\\ \; & \;+|E\eta \tau I_{\left[|\eta |\geq N,|\tau |\geq N\right]}-E\eta I_{\left[|\eta |\geq N\right]}E\tau I_{\left[|\tau |\geq N\right]}|. \end{array}$$

Multiplying and dividing the first term by $N^{2}$ and applying Lemma B.1.2, we get $|E\eta \tau I_{\left[|\eta |\leq N,|\tau |\leq N\right]}-E\eta I_{\left[|\eta |\leq N\right]}E\tau I_{\left[|\tau |\leq N\right]}|\leq 4N^{2}\alpha_{k}$. For the second term, if |τ|≤N, then τ≤N and τ≥−N. Plugging this in the second term we get,

(A18)|EητI[|η|≥N,|τ|≤N]−EηI[|η|≥N]EτI[|τ|≤N]|≤NEηI[|η|≥N+NEηI[|η|≥N](A19)=2N|EηI[|η|≥N]|.

Since |η|≥N, we have $1\leq \frac{{\left|\eta \right|}^{1+\delta }}{N^{1+\delta }}$. Following this,

(A20)|2NEηI[|η|≥N]|≤2NE|η|2+δN1+δI[|η|≥N](A21)≤2N1N1+δ|Eη2+δ|≤2C1Nδ.

Similarly, we can also write for the third term, $|E\eta \tau I_{\left[|\eta |\leq N,|\tau |\geq N\right]}-E\eta I_{\left[|\eta |\leq N\right]}E\tau I_{\left[|\tau |\geq N\right]}|\leq 2\frac{C_{2}}{N^{\delta }}$. Finally, for the last term we get that by Cauchy-Schwarz inequality,

(A22) $$\begin{array}{cc} |E\eta \tau I_{\left[|\eta |\geq N,|\tau |\geq N\right]}-E\eta I_{\left[|\eta |\geq N\right]}E\tau I_{\left[|\tau |\geq N\right]}| & \leq \sqrt{\mathrm{Var}\left[\eta I_{\left[|\eta |\geq N\right]}\right]\mathrm{Var}\left[\tau I_{\left[|\tau |\geq N\right]}\right]} \end{array}$$

(A23) $$\begin{array}{cc} \; & <2\sqrt{\mathrm{Var}\left[\eta I_{\left[|\eta |\geq N\right]}\right]\mathrm{Var}\left[\tau I_{\left[|\tau |\geq N\right]}\right]} \end{array}$$

(A24) $$\begin{array}{cc} \; & \leq 2\sqrt{E\left[\eta^{2}I_{\left[|\eta |\geq N\right]}\right]E\left[\tau^{2}I_{\left[|\tau |\geq N\right]}\right]}. \end{array}$$

Since |η|>N, $1<\frac{{\left|\eta \right|}^{\delta }}{N^{\delta }}$. Similarly, $1<\frac{{\left|\tau \right|}^{\delta }}{N^{\delta }}$. Plugging these in the previous equation, we get,

(A25) $$\begin{array}{cc} \sqrt{E\left[\eta^{2}I_{\left[|\eta |\geq N\right]}\right]E\left[\tau^{2}I_{\left[|\tau |\geq N\right]}\right]} & \leq \sqrt{\frac{1}{N^{2\delta }}E\left[{\left|\eta \right|}^{2+\delta }I_{\left[|\eta |\geq N\right]}\right]E\left[{\left|\tau \right|}^{2+\delta }I_{\left[|\tau |\geq N\right]}\right]} \end{array}$$

(A26) $$\begin{array}{cc} \; & \leq \frac{1}{N^{\delta }}\sqrt{C_{1}C_{2}}. \end{array}$$

Combining the four upper bounds above, we get,

(A27) $$\begin{array}{cc} \left|E\eta \tau -E\eta E\tau \right| & \leq 4N^{2}\alpha_{k}+\frac{2}{N^{\delta }}\left(C_{1}+C_{2}\right)+\frac{2}{N^{\delta }}\sqrt{C_{1}C_{2}}. \end{array}$$

Now, in particular, setting $N=n^{-1/2}\alpha_{k}^{-1/(2+\delta )}$ it follows that

(A28) $$\begin{array}{cc} \left|E\eta \tau -E\eta E\tau \right| & \leq \frac{4}{n}\alpha_{k}^{\delta /(2+\delta )}+2n^{\delta /2}\alpha_{k}^{\delta /(2+\delta )}\left(C_{1}+C_{2}\right)+2n^{\delta /2}\alpha_{k}^{\delta /(2+\delta )}\sqrt{C_{1}C_{2}} \end{array}$$

(A29) $$\begin{array}{cc} \; & =\left(\frac{4}{n}+2n^{\delta /2}\left(C_{1}+C_{2}\right)+2n^{\delta /2}\sqrt{C_{1}C_{2}}\right)\alpha_{k}^{\delta /(2+\delta )}. \end{array}$$

□

**Lemma** **B.1.4.**
*Let $\left\{X_{t}\right\}$ be an α-mixing Markov chain with mixing coefficient $\alpha_{k}$. Further assume that $E|X_{t}{|}^{2+\delta }\leq C_{1}\;\mathrm{and}\;E{\left|X_{t+k}\right|}^{2+\delta }\leq C_{2}$ for some δ>0. Then, for any t and any n>0*

(A30) $$|\mathrm{Cov}\left(X_{t},X_{t+k}\right)|\leq \left(\frac{4}{n}+2n^{\delta /2}\left(C_{1}+C_{2}\right)+2n^{\delta /2}\sqrt{C_{1}C_{2}}\right)\alpha_{k}^{\delta /(2+\delta )}.$$

**Proof.** Set $\eta =X_{t},\tau =X_{t+k}$ Lemma B.1.3. □

We also need to establish the following technical lemma.

**Lemma** **B.1.5.**
*Let $\left\{X_{t}\right\}$ be an α-mixing Markov Chain with mixing coefficients $\left\{\alpha_{t}\right\}$. Then the process $\left\{Y_{t}\right\}$ where $Y_{t}:=\log\left(\frac{p_{\theta_{0}}\left(X_{t}|X_{t-1}\right)}{p_{\theta }\left(X_{t}|X_{t-1}\right)}\right)$ is also α-mixing with mixing coefficients $\left\{{\tilde{\alpha }}_{t}\right\}$ where ${\tilde{\alpha }}_{t}=\alpha_{t-1}$.*

**Proof.** By $Z_{i}$ denote the paired random measure $\left(X_{i},X_{i-1}\right)$. Let $M_{i}^{j}$ denote the sigma field generated by the measures $X_{k},\;\mathrm{where}\;i\leq k\leq j$. By $G_{i}^{j}$ denote the sigma field generated by the measures $Z_{k},\;\mathrm{where}\;i\leq k\leq j$. Let $C\in M_{i-1}^{j}$. Then, *C* can be expressed as $\left(C_{i-1}\times C_{i}\times \cdots \times C_{j}\right)$. for $C_{i-1}\in M_{i-1}^{i-1},\;C_{i}\in M_{i}^{i}\cdots$ and so on. Now, consider a map. $T_{i}^{j}:\left(C_{i-1}\times C_{i}\times \cdots \times C_{j}\right)\overset{}{\to }\left(C_{i-1}\times C_{i}\times C_{i}\times \cdots \times C_{j-1}\times C_{j-1}\times C_{j}\right)$. Note that, $T_{i}^{j}\left(C\right)\in G_{i}^{j}$. It is easy to see that $G_{i}^{j}=T_{i}^{j}\left(M_{i-1}^{j}\right)\cup M_{i-1}^{*j}$, where $T_{i}^{j}\left(M_{i-1}^{j}\right)$ is obtained by applying the map $T_{i}^{j}$ to each element of $M_{i-1}^{j}$. If we assume this latter set to be the range and $M_{i-1}^{j}$ to be the domain, then, by construction, $T_{i}^{j}$ is a bijection. Furthermore, the two classes are made of disjoint sets, i.e., if $A\in T_{i}^{j}\left(M_{i-1}^{j}\right)\;\mathrm{and}\;A^{*}\in M_{i-1}^{*j}$, then $A\cap A^{*}=\phi$. Furthermore, note that $M_{i-1}^{j*}$ is made of impossible sets. i.e., $P\left(A^{*}\right)=0\;\forall \;A^{*}\in M_{i-1}^{j*}$. Now consider the α-mixing coefficients for $Z_{i}$. By definition, it is given by

$$\begin{array}{cc} \alpha_{k}^{z} & =\underset{i}{\sup}\underset{A\in G_{-\infty }^{i},B\in G_{i+k}^{\infty }}{\sup}\left|P\left(A\cap B\right)-P\left(A\right)P\left(B\right)\right|\\ \; & =\underset{i}{\sup}\underset{A\in G_{-\infty }^{i},B\in G_{i+k}^{\infty }}{\sup}\left|P\left(\left(A^{o}\cup A^{*}\right)\cap \left(B^{o}\cup B^{*}\right)\right)-P\left(\left(A^{o}\cup A^{*}\right)\right)P\left(\left(B^{o}\cup B^{*}\right)\right)\right|. \end{array}$$

where,

$A=(A^{o}\cup A^{*})$	$B=(B^{o}\cup B^{*})$
$A^{o}\in T_{-\infty }^{i}\left(M_{-\infty }^{i}\right)$	$A^{*}\in M_{-\infty }^{*i}$
$B^{o}\in T_{i+k-1}^{\infty }\left(M_{j+k-1}^{\infty }\right)$	$B^{*}\in M_{j+k-1}^{*\infty }.$

Then, the expression for the α-mixing coefficient can be reduced into

$$\begin{array}{cc} \alpha_{k}^{z} & =\underset{i}{\sup}\underset{A^{o}\in T_{-\infty }^{i}\left(M_{-\infty }^{i}\right),B^{o}\in T_{i+k-1}^{\infty }\left(M_{i+k-1}^{\infty }\right)}{\sup}\left|P\left(A^{o}\cap B^{o}\right)-P\left(A^{o}\right)P\left(B^{o}\right)\right|. \end{array}$$

Note that, by bijection property of $T_{i}^{j}$, we can find $A^{\prime }\in M_{-\infty }^{i}$ and $B^{\prime }\in M_{i+k-1}^{\infty }$ such that

$$\begin{array}{cc} \alpha_{k}^{z} & =\underset{i}{\sup}\underset{A^{\prime }\in M_{-\infty }^{i},B^{\prime }\in M_{i+k-1}^{\infty }}{\sup}\left|P\left(T_{-\infty }^{i}\left(A^{\prime }\right)\cap T_{i+k-1}^{\infty }\left(B^{\prime }\right)\right)-P\left(T_{-\infty }^{i}\left(A^{\prime }\right)\right)P\left(T_{i+k-1}^{\infty }\left(B^{\prime }\right)\right)\right|.\\ \; & =\alpha_{k-1}. \end{array}$$

Now, $\log\left(\frac{p_{\theta_{0}}\left(X_{n}|X_{n-1}\right)}{p_{\theta }\left(X_{n}|X_{n-1}\right)}\right)$ is just a function of the paired Markov chain $Z_{i}$, therefore it has α-mixing coefficient $\alpha_{k-1}$. □

We now proceed to the proof of Proposition 3. Let $\left\{X_{k}\right\}$ be a stationary α-mixing Markov chain under $\theta_{1}$ with mixing coefficients $\left\{\alpha_{k}\right\}$. Observe that the log-likelihood can be expressed as

$$\begin{array}{cc} r_{n}\left(\theta_{2},\theta_{1}\right) & =\sum_{i=1}^{n}\log\left(\frac{p_{\theta_{1}}\left(X_{i}|X_{i-1}\right)}{p_{\theta_{2}}\left(X_{i}|X_{i-1}\right)}\right)+\log\left(\frac{q_{1}^{\left(0\right)}\left(X_{0}\right)}{q_{2}^{\left(0\right)}\left(X_{0}\right)}\right)\\ \; & \equiv \sum_{i=1}^{n}Y_{i}+Z_{0}. \end{array}$$

Therefore, the variance of the log-likelihood ratio is simply

$$\begin{array}{cc} {\mathrm{Var}}_{\theta_{1}}\left[r_{n}\left(\theta_{2},\theta_{1}\right)\right] & ={\mathrm{Var}}_{\theta_{1}}\left[\sum_{i=1}^{n}Y_{i}+Z_{0}\right]\\ \; & =\sum_{i,j=1}^{n}{\mathrm{Cov}}_{\theta_{1}}\left(Y_{i},Y_{j}\right)+\sum_{i,j=1}^{n}{\mathrm{Cov}}_{\theta_{1}}\left(Y_{i},Z_{0}\right)+{\mathrm{Cov}}_{\theta_{1}}\left(Z_{0},Z_{0}\right). \end{array}$$

It follows from Lemma B.1.5 that $\left\{Y_{k}\right\}$ is a stochastic process with α-mixing coefficients $\alpha_{k-1}$. Therefore, using Lemma B.1.4 we have

$$\begin{array}{cc} |{\mathrm{Cov}}_{\theta_{1}}\left(Y_{i},Y_{j}\right)| & =|E_{\theta_{1}}Y_{i}Y_{j}-E_{\theta_{1}}Y_{i}E_{\theta_{1}}Y_{j}|\\ \; & <(\frac{4}{n}+2n^{\delta /2}(E_{\theta_{1}}|Y_{i}{|}^{2+\delta }+E_{\theta_{1}}{\left|Y_{j}\right|}^{2+\delta }\\ \; & \;+\sqrt{E_{\theta_{1}}|Y_{i}{|}^{2+\delta }E_{\theta_{1}}{\left|Y_{j}\right|}^{2+\delta }}))\alpha_{|j-i|-1}^{\delta /(2+\delta )}\\ \; & =\left(\frac{4}{n}+2n^{\delta /2}\left(C_{\theta_{1},\theta_{2}}^{\left(i\right)}+C_{\theta_{1},\theta_{2}}^{\left(j\right)}+\sqrt{C_{\theta_{1},\theta_{2}}^{\left(i\right)}C_{\theta_{1},\theta_{2}}^{\left(j\right)}}\right)\right)\alpha_{|j-i|-1}^{\delta /(2+\delta )}. \end{array}$$

Similarly, as above we can also say

$$\begin{array}{cc} |{\mathrm{Cov}}_{\theta_{1}}\left(Y_{i},Z_{0}\right)|< & \left(\frac{4}{n}+2n^{\delta /2}\left(C_{\theta_{1},\theta_{2}}^{\left(i\right)}+D_{1,2}+\sqrt{C_{\theta_{1},\theta_{2}}^{\left(i\right)}D_{1,2}}\right)\right)\left(\alpha_{i-1}^{\delta /(2+\delta )}\right) \end{array}$$

Combining, the two upper bounds above, we get the first result:

$$\begin{array}{cc} {\mathrm{Var}}_{\theta_{1}}\left[r_{n}\left(\theta_{2},\theta_{1}\right)\right] & <\sum_{i,j=1}^{n}\left(\frac{4}{n}+2n^{\delta /2}\left(C_{\theta_{1},\theta_{2}}^{\left(i\right)}+C_{\theta_{1},\theta_{2}}^{\left(j\right)}+\sqrt{C_{\theta_{1},\theta_{2}}^{\left(i\right)}C_{\theta_{1},\theta_{2}}^{\left(j\right)}}\right)\right)\left(\alpha_{|i-j|-1}^{\delta /(2+\delta )}\right)\\ \; & \;+\sum_{i=1}^{n}\left(\frac{4}{n^{2}}+2n^{\delta /2}\left(C_{\theta_{1},\theta_{2}}^{\left(i\right)}+D_{1,2}+\sqrt{C_{\theta_{1},\theta_{2}}^{\left(i\right)}D_{1,2}}\right)\right)\left(\alpha_{i-1}^{\delta /(2+\delta )}\right)\\ \; & \;+\mathrm{Var}[Z_{0},Z_{0}]. \end{array}$$

If $\left\{X_{i}\right\}$ is stationary under $\theta_{1}$, so is $\left\{Y_{i}\right\}$. Therefore, $E_{\theta_{1}}|Y_{i}{|}^{2+\delta }=E_{\theta_{1}}{\left|Y_{1}\right|}^{2+\delta }=C_{\theta_{1},\theta_{2}}^{\left(1\right)}\;\forall \;i$, and

(A31) $$\begin{array}{cc} \sum_{i,j=1}^{n}{\mathrm{Cov}}_{\theta_{1}}\left(Y_{i},Y_{j}\right) & \leq \sum_{i,j=1}^{n}\left(\frac{4}{n}+6n^{\delta /2}C_{\theta_{1},\theta_{2}}^{\left(1\right)}\right)\alpha_{|j-i|-1}^{\delta /(2+\delta )}\\ \; & \leq n\;\left(\frac{4}{n}+6n^{\delta /2}C_{\theta_{1},\theta_{2}}^{\left(1\right)}\right)\left(\sum_{h\geq 1}\alpha_{h-1}^{\delta /(2+\delta )}\right). \end{array}$$

Again, using Lemma B.1.4 on ${\mathrm{Cov}}_{\theta_{1}}\left(Y_{i},Z_{0}\right)$, yields

(A32) $$\sum_{i=1}^{n}{\mathrm{Cov}}_{\theta_{1}}\left(Y_{i},Z_{0}\right)\leq \left(\frac{4}{n}+2n^{\delta /2}\left(C_{\theta }+D_{1,2}+\sqrt{C_{\theta }D_{1,2}}\right)\right)\left(\sum_{h\geq 1}\alpha_{h}^{\delta /(2+\delta )}\right).$$

Finally, using Equations (A31) and (A32) we have

$$\begin{array}{cc} {\mathrm{Var}}_{\theta_{1}}\left[r_{n}\left(\theta_{2},\theta_{1}\right)\right] & \leq n\left(\frac{4}{n}+6n^{\delta /2}C_{\theta_{1},\theta_{2}}^{\left(1\right)}\right)\left(\sum_{h\geq 1}\alpha_{h-1}^{\delta /(2+\delta )}\right)+\\ \; & \left(\frac{4}{n}+2n^{\delta /2}\left(C_{\theta_{1},\theta_{2}}^{\left(1\right)}+D_{1,2}+\sqrt{C_{\theta_{1},\theta_{2}}^{\left(1\right)}D_{1,2}}\right)\right)\left(\sum_{h\geq 1}\alpha_{h}^{\delta /(2+\delta )}\right)\\ \; & \;+{\mathrm{Cov}}_{\theta_{1}}\left(Z_{0},Z_{0}\right). \end{array}$$

□

### Appendix B.2. Proofs for Stationary Markov Data-Generating Models

#### Proof of Theorem 2

*Part 1: Verifying condition (i) of Corollary 1.*

We substitute the true parameter $\theta_{0}$ for $\theta_{1}$ and θ for $\theta_{2}$. We also set $q_{1}^{\left(0\right)}$ to be the invariant distribution of the Markov chain under $\theta_{0}$, $q_{0}$, and $q_{2}^{\left(0\right)}$ as the invariant distribution of the Markov chain under θ, $q_{\theta }$. Applying the fact that these Markov chains are stationary to Proposition 2, we have

(A33) $$\begin{array}{cc} K(P_{\theta_{0}}^{\left(n\right)},P_{\theta }^{\left(n\right)})\; & =nE\left[\log\left(\frac{p_{\theta_{0}}\left(X_{1}|X_{0}\right)}{p_{\theta }\left(X_{1}|X_{0}\right)}\right)\right]+E\left[Z_{0}\right],\\ \; & \leq n\sum_{j=1}^{m}E\left[M_{j}^{\left(1\right)}\left(X_{1},X_{0}\right)\right]\left|f_{j}^{\left(1\right)}\left(\theta ,\theta_{0}\right)\right|+\sum_{k=1}^{m}E\left[M_{k}^{\left(2\right)}\left(X_{0}\right)\right]\left|f_{k}^{\left(2\right)}\left(\theta ,\theta_{0}\right)\right|, \end{array}$$

where the inequality follows from Assumption 1. Therefore, it follows that

$$\begin{array}{cc} \int K\left(P_{\theta_{0}}^{\left(n\right)},P_{\theta }^{\left(n\right)}\right)\rho_{n}\left(d\theta \right) & \leq n\sum_{j=1}^{m}E\left[M_{j}^{\left(1\right)}\left(X_{1},X_{0}\right)\right]\int \left|f_{j}^{\left(1\right)}\left(\theta ,\theta_{0}\right)\right|\rho_{n}\left(d\theta \right)\\ \; & +\sum_{k=1}^{m}E\left[M_{k}^{\left(2\right)}\left(X_{0}\right)\right]\left|\int f_{k}^{\left(2\right)}\left(\theta ,\theta_{0}\right)\right|\rho_{n}\left(d\theta \right). \end{array}$$

By Assumption 1(i), it follows that

$$\begin{array}{cc} \int K\left(P_{\theta_{0}}^{\left(n\right)},P_{\theta }^{\left(n\right)}\right)\rho_{n}\left(d\theta \right) & \leq n\sum_{j=1}^{m}E\left[M_{j}^{\left(1\right)}\left(X_{1},X_{0}\right)\right]\frac{C}{\sqrt{n}}+\sum_{k=1}^{m}E\left[M_{k}^{\left(2\right)}\left(X_{0}\right)\right]\frac{C}{\sqrt{n}}\leq n\epsilon_{n}^{\left(1\right)}, \end{array}$$

where $\epsilon_{n}^{\left(1\right)}=O\left(\frac{1}{\sqrt{n}}\right)$.

*Part 2: Verifying condition (ii) of 1.* Again, using Proposition 3 along with the fact that the Markov chain is stationary we have

$$\begin{array}{cc} \mathrm{Var}[r_{n}\left(\theta ,\theta_{0}\right)] & \leq n\left(\frac{4}{n}+6n^{\delta /2}C_{\theta_{0},\theta }^{\left(1\right)}\right)\left(\sum_{k\geq 0}\alpha_{k}^{\delta /(2+\delta )}\right)\\ \; & \;+\left(\frac{4}{n^{2}}+2n^{\delta /2}\left(C_{\theta_{0},\theta }^{\left(1\right)}+D_{\theta_{0},\theta }+\sqrt{C_{\theta_{0},\theta }^{\left(1\right)}D_{\theta_{0},\theta }}\right)\right)\left(\sum_{k\geq 1}\alpha_{k}^{\delta /(2+\delta )}\right)\\ \; & \;+\mathrm{Var}[Z_{0}]. \end{array}$$

It then follows that

$$\begin{array}{cc} \int \mathrm{Var}\left[r_{n}\left(\theta ,\theta_{0}\right)\right]\rho_{n}\left(d\theta \right) & \leq n\left(\frac{4}{n}+6n^{\delta /2}\int C_{\theta_{0},\theta }^{\left(1\right)}\rho_{n}\left(d\theta \right)\right)\left(\sum_{k\geq 1}\alpha_{k-1}^{\delta /(2+\delta )}\right)+\int \mathrm{Var}\left[Z_{0}\right]\rho_{n}\left(d\theta \right)\\ \; & \;+(\frac{4}{n^{2}}+2n^{\delta /2}(\int C_{\theta_{0},\theta }^{\left(1\right)}\rho_{n}\left(d\theta \right)\\ \; & \;\;+\int D_{\theta_{0},\theta }\rho_{n}\left(d\theta \right)+\int \sqrt{C_{\theta_{0},\theta }^{\left(1\right)}D_{\theta_{0},\theta }}\rho_{n}\left(d\theta \right)))\left(\sum_{k\geq 1}\alpha_{k}^{\delta /(2+\delta )}\right). \end{array}$$

First, consider the term $\int C_{\theta_{0},\theta }^{\left(1\right)}\rho_{n}\left(\theta \right)$, and observe that

$$\begin{array}{cc} \int C_{\theta_{0},\theta }^{\left(1\right)}\rho_{n}\left(d\theta \right) & =\int E\log{\left|\frac{p_{\theta_{0}}\left(X_{1}|X_{0}\right)}{p_{\theta }\left(X_{1}|X_{0}\right)}\right|}^{2+\delta }\rho_{n}\left(d\theta \right). \end{array}$$

By Assumption 1, we have

$$\begin{array}{cc} \int E\log{\left|\frac{p_{\theta_{0}}\left(X_{1}|X_{0}\right)}{p_{\theta }\left(X_{1}|X_{0}\right)}\right|}^{2+\delta }\rho_{n}\left(d\theta \right) & \leq \int E{\left[\sum_{j=1}^{m}M_{j}^{\left(1\right)}\left(X_{1},X_{0}\right)\left|f_{k}^{\left(1\right)}\left(\theta ,\theta_{0}\right)\right|\right]}^{2+\delta }\rho_{n}\left(d\theta \right). \end{array}$$

Since the function $x\mapsto x^{2+\delta }$ is convex, we can apply Jensen’s inequality to obtain,

$$\begin{array}{cc} {\left(\sum_{j=1}^{m}M_{j}^{\left(1\right)}\left(X_{1},X_{0}\right)\left|f_{k}^{\left(1\right)}\left(\theta ,\theta_{0}\right)\right|\right)}^{2+\delta } & \leq m^{1+\delta }\sum_{k=1}^{m}M_{j}^{\left(1\right)}{\left(X_{1},X_{0}\right)}^{2+\delta }{\left|f_{k}^{\left(1\right)}\left(\theta ,\theta_{0}\right)\right|}^{2+\delta }. \end{array}$$

Therefore, it follows that

$$\begin{array}{cc} \int E\log{\left|\frac{p_{\theta_{0}}\left(X_{1}|X_{0}\right)}{p_{\theta }\left(X_{1}|X_{0}\right)}\right|}^{2+\delta }\rho_{n}\left(d\theta \right) & \leq m^{1+\delta }\sum_{k=1}^{m}E\left[M_{k}^{\left(1\right)}{\left(X_{1},X_{0}\right)}^{2+\delta }\right]\\ \; & \;\times \int |f_{k}^{\left(1\right)}\left(\theta ,\theta_{0}\right){|}^{2+\delta }\rho_{n}\left(d\theta \right). \end{array}$$

By Assumption 1, $\int |f_{k}\left(\theta ,\theta_{0}\right){|}^{2+\delta }\rho_{n}\left(d\theta \right)<\frac{C}{n}$ and $E[M_{k}^{\left(1\right)}{\left(X_{1},X_{0}\right)}^{2+\delta }]<B$, implying that

$$\begin{array}{cc} \int C_{\theta_{0},\theta }^{\left(1\right)}\rho_{n}\left(d\theta \right) & \leq m^{1+\delta }\sum_{k=1}^{m}B\frac{C}{n}=m^{2+\delta }\frac{BC}{n}. \end{array}$$

Since $\left(\sum_{k\geq 0}\alpha_{k}^{\delta /(2+\delta )}\right)<\infty$, it follows that $\left(\frac{4}{n}+6n^{\delta /2}\int C_{\theta_{0},\theta }^{\left(1\right)}\rho_{n}\left(d\theta \right)\right)\left(\sum_{k\geq 1}\alpha_{k-1}^{\delta /(2+\delta )}\right)=O\left(\frac{n^{\delta /2}}{n}\right)$. Similarly, we can show that $\int D_{\theta_{0},\theta }\rho_{n}\left(d\theta \right)=O\left(\frac{1}{n}\right)$, and $\int \mathrm{Var}\left[Z_{0}\right]\rho_{n}\left(d\theta \right)=O\left(\frac{1}{n}\right)$.

For the final term $\int \sqrt{C_{\theta_{0},\theta }^{\left(1\right)}D_{\theta_{0},\theta }}\rho_{n}\left(d\theta \right)$, use the Cauchy-Schwarz inequality to obtain the upper bound ${\left(\int C_{\theta_{0},\theta }^{\left(1\right)}\rho_{n}\left(d\theta \right)\int D_{\theta_{0},\theta }\rho_{n}\left(d\theta \right)\right)}^{1/2}$ which is also of order $O(\frac{1}{n})$. Combining all of these together we have

$$\begin{array}{c} \int \mathrm{Var}\left[r_{n}\left(\theta ,\theta_{0}\right)\right]\rho_{n}\left(d\theta \right)\leq n\epsilon_{n}^{\left(2\right)}, \end{array}$$

for some $\epsilon_{n}^{\left(2\right)}=O\left(\frac{n^{\delta /2}}{n}\right)$.

Since $K\left(\rho_{n},\pi \right)<\sqrt{n}C=n\frac{C}{\sqrt{n}},$ it follows that $K\left(\rho_{n},\pi \right)<n\epsilon_{n}^{\left(3\right)},$ where $\epsilon_{n}^{\left(3\right)}=O\left(1/\sqrt{n}\right)$ as before. Finally, by choosing $\epsilon_{n}=\max\left(\epsilon_{n}^{\left(1\right)},\epsilon_{n}^{\left(2\right)},\epsilon_{n}^{\left(3\right)}\right)$, our theorem is proved. □

### Appendix B.3. Proofs for Non-Stationary, Ergodic Markov Data-Generating Models

#### Appendix B.3.1. Proof of Theorem 3

*Part 1: Verifying condition (i) of Corollary 1:* As in the proof of Theorem 2 substitute the true parameter $\theta_{0}$ for $\theta_{1}$ and θ for $\theta_{2}$ in. We also set $q_{1}^{\left(0\right)}$ and $q_{2}^{\left(0\right)}$ to the distribution $q^{\left(0\right)}$. Applying Proposition 2 to the corresponding transition kernels and initial distribution we have,

(A34) $$\begin{array}{cc} K(P_{\theta_{0}}^{\left(n\right)},P_{\theta }^{\left(n\right)}) & =\sum_{i=1}^{n}E\left[\log\left(\frac{p_{\theta_{0}}\left(X_{i}|X_{i-1}\right)}{p_{\theta }\left(X_{i}|X_{i-1}\right)}\right)\right]+E\left[\log\left(\frac{D(X_{0})}{D(X_{0})}\right)\right]\\ \; & =\sum_{i=1}^{n}E\left[\log\left(\frac{p_{\theta_{0}}\left(X_{i}|X_{i-1}\right)}{p_{\theta }\left(X_{i}|X_{i-1}\right)}\right)\right]. \end{array}$$

Now, applying Assumption 1, we can bound the previous equation as follows,

(A35) $$\begin{array}{cc} \;K(P_{\theta_{0}}^{\left(n\right)},P_{\theta }^{\left(n\right)}) & \leq \sum_{i=1}^{n}E\left[\sum_{k=1}^{m}M_{k}^{\left(1\right)}\left(X_{i},X_{i-1}\right)\left|f_{k}^{\left(1\right)}\left(\theta ,\theta_{0}\right)\right|\right]\\ \; & =\sum_{i=1}^{n}\sum_{k=1}^{m}E\left[M_{k}^{\left(1\right)}\left(X_{i},X_{i-1}\right)\right]\left|f_{k}^{\left(1\right)}\left(\theta ,\theta_{0}\right)\right|. \end{array}$$

Since $M_{k}^{\left(1\right)}$’s are bounded there exists a constant *Q* so that,

$$\begin{array}{cc} \int K\left(P_{\theta_{0}}^{\left(n\right)},P_{\theta }^{\left(n\right)}\right)\rho_{n}\left(d\theta \right) & \leq Q\int \sum_{i=1}^{n}\sum_{k=1}^{m}\left|f_{k}^{\left(1\right)}\left(\theta ,\theta_{0}\right)\right|\rho_{n}\left(d\theta \right)\\ \; & =Qn\sum_{k=1}^{m}\int \left|f_{k}^{\left(1\right)}\left(\theta ,\theta_{0}\right)\right|\rho_{n}\left(d\theta \right). \end{array}$$

By Assumption 19 in Assumption 1, it follows that

$$\begin{array}{cc} \int K\left(P_{\theta_{0}}^{\left(n\right)},P_{\theta }^{\left(n\right)}\right)\rho_{n}\left(d\theta \right) & \leq Qn\sum_{k=1}^{m}\frac{C}{\sqrt{n}}=nmQ\frac{C}{\sqrt{n}}=n\epsilon_{n}^{\left(1\right)}, \end{array}$$

for some $\epsilon_{n}^{\left(1\right)}=O\left(\frac{1}{\sqrt{n}}\right)$.

*Part 2: Verifying condition (ii) of Corollary 1:* As in the previous part, $Z_{0}=0$, implying that $D_{\theta ,\theta_{0}}$. Applying Proposition 3 and integrating with respect to $\rho_{n}$, we obtain

(A36) $$\begin{array}{cc} \int \mathrm{Var}\left[r_{n}\left(\theta ,\theta_{0}\right)\right]\rho_{n}\left(d\theta \right)\; & \leq \sum_{i=1}^{n}\left(\frac{4}{n}+2n^{\delta /2}\int C_{\theta_{0},\theta }^{\left(i\right)}\rho_{n}\left(d\theta \right)\right)\left(\alpha_{i-1}^{\delta /(2+\delta )}\right)\\ \; & \;+\sum_{i,j=1}^{n}\left(\frac{4}{n}+2n^{\delta /2}\left(\int C_{\theta_{0},\theta }^{\left(i\right)}\rho_{n}\left(d\theta \right)+\int C_{\theta_{0},\theta }^{\left(j\right)}\rho_{n}\left(d\theta \right)+\int \sqrt{C_{\theta_{0},\theta }^{\left(i\right)}C_{\theta_{0},\theta }^{\left(j\right)}}\rho_{n}\left(d\theta \right)\right)\right)\\ \; & \;\times \left(\alpha_{|i-j|-1}^{\delta /(2+\delta )}\right). \end{array}$$

First, consider the term $\int C_{\theta_{0},\theta }^{\left(i\right)}\rho_{n}\left(d\theta \right)$. Using Assumption 1, we can upper bound $C_{\theta_{0},\theta }^{\left(i\right)}$ as,

$$\begin{array}{cc} C_{\theta_{0},\theta }^{\left(i\right)} & \leq E{\left[\sum_{k=1}^{m}M_{k}^{\left(1\right)}\left(X_{i},X_{i-1}\right)\left|f_{k}^{\left(1\right)}\left(\theta ,\theta_{0}\right)\right|\right]}^{2+\delta }\\ \; & \leq \sum_{k=1}^{m}m^{1+\delta }E\left[{\left(M_{k}^{\left(1\right)}\left(X_{i},X_{i-1}\right)\left|f_{k}^{\left(1\right)}\left(\theta ,\theta_{0}\right)\right|\right)}^{2+\delta }\right](\mathrm{by}\;\mathrm{Jensen}’s\;\mathrm{inequality})\\ \; & =\sum_{k=1}^{m}m^{1+\delta }E\left[M_{k}^{\left(1\right)}{\left(X_{i},X_{i-1}\right)}^{2+\delta }\right]{\left|f_{k}^{\left(1\right)}\left(\theta ,\theta_{0}\right)\right|}^{2+\delta }. \end{array}$$

Since $M_{k}^{\left(1\right)}$’s are upper bounded by *Q*, it follows from the previous expression that, $C_{\theta_{0},\theta }^{\left(i\right)}\leq \sum_{k=1}^{m}m^{1+\delta }Q^{2+\delta }{\left|f_{k}^{\left(1\right)}\left(\theta ,\theta_{0}\right)\right|}^{2+\delta }$.

Hence, from Assumption 1, we get,

$$\begin{array}{cc} \int C_{\theta_{0},\theta }^{\left(i\right)}\rho_{n}\left(d\theta \right) & \leq \sum_{k=1}^{m}m^{1+\delta }Q^{2+\delta }\int {\left|f_{k}^{\left(1\right)}\left(\theta ,\theta_{0}\right)\right|}^{2+\delta }\rho_{n}\left(d\theta \right)\leq {\left(mQ\right)}^{2+\delta }\frac{C}{n}. \end{array}$$

Using the upper bound above, we can say for an *L* large enough, $\int C_{\theta_{0},\theta }^{\left(i\right)}\rho_{n}\left(d\theta \right)\leq \frac{L}{n}$. Next, by the Cauchy-Schwarz inequality, we have that $\int \sqrt{C_{\theta_{0},\theta }^{\left(i\right)}C_{\theta_{0},\theta }^{\left(j\right)}\rho_{n}\left(d\theta \right))}<\sqrt{\int C_{\theta_{0},\theta }^{\left(i\right)}\rho_{n}\left(d\theta \right)\int C_{\theta_{0},\theta }^{\left(j\right)}\rho_{n}\left(d\theta \right))}\leq \frac{L}{n}$. Thus, we have the following upper bound.

$$\begin{array}{cc} \int \mathrm{Var}\left[r_{n}\left(\theta ,\theta_{0}\right)\right]\rho_{n}\left(d\theta \right) & \leq \sum_{i=1}^{n}\left(\frac{4}{n}+2n^{\delta /2}\frac{L}{n}\right)\left(\alpha_{i-1}^{\delta /(2+\delta )}\right)\\ \; & \;+\sum_{i,j=1}^{n}\left(\frac{4}{n}+2n^{\delta /2}\left(\frac{L}{n}+\frac{L}{n}+\frac{L}{n}\right)\right)\left(\alpha_{|i-j|-1}^{\delta /(2+\delta )}\right)\\ \; & =\left(\frac{4}{n}+2n^{\delta /2}\frac{L}{n}\right)\left(\sum_{i=1}^{n}\alpha_{i-1}^{\delta /(2+\delta )}\right)\\ \; & \;+\left(\frac{4}{n}+6n^{\delta /2}\frac{L}{n}\right)\left(\sum_{i,j=1}^{n}\alpha_{|i-j|-1}^{\delta /(2+\delta )}\right). \end{array}$$

Since $\sum_{i,j=1}^{n}\alpha_{|i-j|-1}^{\delta /(2+\delta )}<n\sum_{k\geq 1}\alpha_{k-1}^{\delta /(2+\delta )}<\infty$, we have that for some $\epsilon_{n}^{\left(2\right)}=O\left(\frac{n^{\delta /2}}{n}\right)$,

$$\begin{array}{c} \int \mathrm{Var}\left[r_{n}\left(\theta ,\theta_{0}\right)\right]\rho_{n}\left(d\theta \right)<n\epsilon_{n}^{\left(2\right)}. \end{array}$$

Since $K\left(\rho_{n},\pi \right)\leq \sqrt{n}C$, following the concluding argument in Theorem 2 completes the proof. □

#### Appendix B.3.2. Proof of Proposition 8

We verify Assumption 1 and the proof follows from Theorem 3. For i∈{1,2,⋯,K−1},

$$\begin{array}{c} p_{\theta }\left(j|i\right)=\left\{\begin{array}{cc} \theta & \;\mathrm{if}\;j=i-1,\\ 1-\theta & \;\mathrm{if}\;j=i+1. \end{array}\right. \end{array}$$

If i=0 or i=K, then the Markov chain goes back to 1 or K−1, respectively, with probability 1. With the convention $\log\frac{0}{0}=0$, the log ratio of the transition probabilities becomes,

$$\begin{array}{c} |\log p_{\theta_{0}}\left(X_{1}|X_{0}\right)-\log p_{\theta }\left(X_{1}|X_{0}\right)|=I_{\left[X_{1}=X_{0}+1\right]}\log\left(\frac{\theta_{0}}{\theta }\right)+I_{\left[X_{1}=X_{0}-1\right]}\log\left(\frac{1-\theta_{0}}{1-\theta }\right). \end{array}$$

In this case, m=2. $M_{1}^{\left(1\right)}\left(X_{1},X_{0}\right)=I_{\left[X_{1}=X_{0}+1\right]}$ and $M_{2}^{\left(1\right)}\left(X_{1},X_{0}\right)=I_{\left[X_{1}=X_{0}-1\right]}$, both of which are bounded. Let $f_{1}^{\left(1\right)}\left(\theta ,\theta_{0}\right):=\log\left(\frac{\theta_{0}}{\theta }\right)$ suppose $f_{2}^{\left(1\right)}\left(\theta ,\theta_{0}\right):=\log\left(\frac{1-\theta_{0}}{1-\theta }\right)$.

The stationary distribution $q_{\theta }\left(i\right)=\frac{1}{K}\;\forall \;i\in 1,2,\cdots ,K$. Hence the log of the ratio of the invariant distribution becomes

(A37) $$\begin{array}{cc} \log q_{0}\left(x\right)-\log q_{\theta }\left(x\right) & =0, \end{array}$$

and we can set $M_{i}^{\left(2\right)}\left(\cdot \right):=1$ and $f_{i}^{\left(2\right)}\left(\cdot ,\cdot \right):=0$ for i∈{1,2}. Thus, to prove the concentration bound for this Markov chain it is enough to assume that δ=1 and show that $\int {\left[f_{1}^{\left(1\right)}\left(\theta ,\theta_{0}\right)\right]}^{3}\rho_{n}\left(d\theta \right)<\frac{C}{n}$ and $\int {\left[f_{2}^{\left(1\right)}\left(\theta ,\theta_{0}\right)\right]}^{3}\rho_{n}\left(d\theta \right)<\frac{C}{n}$ for some constant C>0.

As given, $\left\{\rho_{n}\right\}$ is a sequence of beta probability distribution functions, with parameters $a_{n},b_{n}$ that satisfy the constraint $\frac{a_{n}}{a_{n}+b_{n}}=\theta_{0}$. Specifically, we choose $a_{n}=n\theta_{0}$ and (therefore) $b_{n}=n\left(1-\theta_{0}\right)$. Thus, we get the following,

$$\begin{array}{cc} \int |f_{1}^{\left(1\right)}\left(\theta ,\theta_{0}\right){|}^{3}\rho_{n}\left(d\theta \right) & =\int {\left|\log\left(\frac{\theta_{0}}{\theta }\right)\right|}^{3}\rho_{n}\left(d\theta \right)\\ \; & <\int {\left|\frac{\theta_{0}}{\theta }-1\right|}^{3}\rho_{n}\left(d\theta \right)\\ \; & =\frac{1}{\mathrm{Beta}(a_{n},b_{n})}\int_{0}^{1}{\left|\frac{\theta_{0}-\theta }{\theta }\right|}^{3}\theta^{a_{n}-1}{\left(1-\theta \right)}^{b_{n}-1}d\theta . \end{array}$$

Since $\theta_{0},\theta \in \left(0,1\right)$, so is $\frac{|\theta_{0}-\theta |}{2}$, giving $|\theta_{0}{-\theta |}^{3}<2{\left(\theta_{0}-\theta \right)}^{2}$. We use that fact to arrive at

$$\begin{array}{cc} \int |f_{1}^{\left(1\right)}\left(\theta ,\theta_{0}\right){|}^{3}\rho_{n}\left(d\theta \right) & \leq \frac{2}{\mathrm{Beta}(a_{n},b_{n})}\int_{0}^{1}{\left(\theta_{0}-\theta \right)}^{2}\theta^{a_{n}-4}{\left(1-\theta \right)}^{b_{n}-1}d\theta \\ \; & =\frac{2\mathrm{Beta}(a_{n}-3,b_{n})}{\mathrm{Beta}(a_{n},b_{n})}\frac{\left(a_{n}-3\right)\left(b_{n}\right)}{{\left(a_{n}+b_{n}-3\right)}^{2}\left(a_{n}+b_{n}-2\right)}. \end{array}$$

From our choice of $a_{n}$ and $b_{n}$, $\frac{2\mathrm{Beta}(a_{n}-3,b_{n})}{\mathrm{Beta}(a_{n},b_{n})}=O\left(1\right)$, and plugging the values of $a_{n}$ and $b_{n}$ into $\frac{\left(a_{n}-3\right)\left(b_{n}\right)}{{\left(a_{n}+b_{n}-3\right)}^{2}\left(a_{n}+b_{n}-2\right)}$, we get $\frac{\left(a_{n}-3\right)\left(b_{n}\right)}{{\left(a_{n}+b_{n}-3\right)}^{2}\left(a_{n}+b_{n}-2\right)}=\frac{1}{n}\frac{\left(\theta_{0}-\frac{3}{n}\right)\left(1-\theta_{0}\right)}{{\left(1-\frac{3}{n}\right)}^{2}\left(1-\frac{2}{n}\right)}$, which is upper bounded by $\frac{C_{1}}{n}$ for some constant $C_{1}>0$. Hence,

$$\begin{array}{cc} \int |f_{1}^{\left(1\right)}\left(\theta ,\theta_{0}\right){|}^{3}\rho_{n}\left(d\theta \right) & <\frac{C_{1}}{n}. \end{array}$$

Similarly, we can also show that,

$$\begin{array}{cc} \int |f_{2}^{\left(1\right)}\left(\theta ,\theta_{0}\right){|}^{3}\rho_{n}\left(d\theta \right) & <\frac{C_{2}}{n}. \end{array}$$

Finally, from Proposition A.2.1, we get that $K\left(\rho_{n},\pi \right)<C+\frac{1}{2}\log\left(n\right)$ for some large constant *C*. Hence, $K\left(\rho_{n},\pi \right)<C_{3}\sqrt{n}$ for some constant $C_{3}>0$. Choosing $C=\max(C_{1},C_{2},C_{3})$, we satisfy all the conditions of Assumption 1 and Theorem 3. □

#### Appendix B.3.3. Proof of Proposition 9

For the purpose of this proof, we choose $\rho_{n}$’s with scaled Beta distribution with parameters $a_{n}=n\left(\theta_{0}/2\right)$ and $b_{n}=n\left(1-\theta_{0}/2\right)$. Since, $\rho_{n}$ is a scaled Beta distribution with the scaling factors m=0.5 and c=0, the pdf of $\rho_{n}$ is given by

$$\begin{array}{cc} \rho_{n}\left(\theta \right) & =\frac{2}{\mathrm{Beta}(a_{n},b_{n})}{\left(2\theta \right)}^{a_{n}}{\left(1-2\theta \right)}^{b_{n}} \end{array}$$

Since this is a scaled distribution, $E_{\rho_{n}}\left[\theta \right]=2\frac{a_{n}}{a_{n}+b_{n}}=\theta_{0}$ and there exists a constant σ>0, ${\mathrm{Var}}_{\rho_{n}}\left[\theta \right]=\frac{\sigma^{2}}{n}$. Now, we analyse the transition probabilities. For i∈{1,2,⋯}, the Birth-Death process has transition probabilities

$$\begin{array}{c} p_{\theta }\left(j|i\right)=\left\{\begin{array}{cc} \theta & \;\mathrm{if}\;j=i-1,\\ 1-\theta & \;\mathrm{if}\;j=i+1. \end{array}\right. \end{array}$$

If i=0, then the Markov chain goes to 1 with probability 1. Hence with the convention $\log\frac{0}{0}=0$ the ratio of the log of the transition probabilities becomes,

$$\begin{array}{c} |\log p_{\theta_{0}}\left(X_{1}|X_{0}\right)-\log p_{\theta }\left(X_{1}|X_{0}\right)|=I_{\left[X_{1}=X_{0}+1\right]}\log\left[\frac{\theta_{0}}{\theta }\right]+I_{\left[X_{1}=X_{0}-1\right]}\log\left[\frac{1-\theta_{0}}{1-\theta }\right]. \end{array}$$

In this case, m=3. $M_{1}^{\left(1\right)}\left(X_{1},X_{0}\right)=I_{\left[X_{1}=X_{0}+1\right]}$ and $M_{2}^{\left(1\right)}\left(X_{1},X_{0}\right)=I_{\left[X_{1}=X_{0}-1\right]}$. Define $M_{3}^{\left(1\right)}\left(X_{1},X_{0}\right):=1$. All these random variables are bounded. Define $f_{1}^{\left(1\right)}\left(\theta ,\theta_{0}\right):=\log\left[\frac{\theta_{0}}{\theta }\right],f_{2}^{\left(1\right)}\left(\theta ,\theta_{0}\right):=\log\left[\frac{1-\theta_{0}}{1-\theta }\right]$ and $f_{3}^{\left(1\right)}\left(\theta ,\theta_{0}\right):=0$. Similarly as in the proof on Proposition 8,

$$\begin{array}{cc} \int {\left[f_{1}^{\left(1\right)}\left(\theta ,\theta_{0}\right)\right]}^{3}\rho_{n}\left(d\theta \right) & <\frac{C_{1}}{n},\;\mathrm{and}\;\\ \int {\left[f_{2}^{\left(1\right)}\left(\theta ,\theta_{0}\right)\right]}^{3}\rho_{n}\left(d\theta \right) & <\frac{C_{2}}{n}. \end{array}$$

The stationary distribution is given by $q_{\theta }\left(i\right)={\left(\frac{\theta }{1-\theta }\right)}^{i-1}q_{\theta }\left(1\right)\;\forall \;i\in 1,2,\cdots$, so that $q_{\theta }\left(i\right)=\left(1-\theta \right){\left(\frac{\theta }{1-\theta }\right)}^{i-1}$ Hence the log of the ratio of the invariant distribution becomes

(A38) $$\begin{array}{cc} \log q_{0}\left(i\right)-\log q_{\theta }\left(i\right) & =\log\left[\frac{1-\theta_{0}}{1-\theta }\right]+\left(i-1\right)\log\left[\frac{\theta_{0}}{\theta }\right]-\left(i-1\right)\log\left[\frac{1-\theta_{0}}{1-\theta }\right] \end{array}$$

We define $M_{1}^{\left(2\right)}\left(X_{0}\right):=1$, and $M_{2}^{\left(2\right)}\left(X_{0}\right)=M_{3}^{\left(2\right)}\left(X_{0}\right):=X_{0}-1$. We can write $E_{q^{\left(0\right)}}{\left[M_{2}^{\left(2\right)}\left(X_{0}\right)\right]}^{2}=\sum_{i=1}^{\infty }{\left(i-1\right)}^{2}q^{\left(0\right)}\left(i\right)<\sum_{i=1}^{\infty }i^{2}q^{\left(0\right)}\left(i\right)$. We have chosen $q^{\left(0\right)}$ such that $\sum_{i=1}^{\infty }i^{2}q^{\left(0\right)}\left(i\right)$ is bounded. Hence, $E_{q^{\left(0\right)}}{\left[M_{2}^{\left(2\right)}\left(X_{0}\right)\right]}^{2}<\infty$. To verify Assumption i define, $f_{1}^{\left(2\right)}\left(\theta ,\theta_{0}\right)=-f_{3}^{\left(2\right)}\left(\theta ,\theta_{0}\right):=\log\left[\frac{1-\theta_{0}}{1-\theta }\right]$, and define $f_{2}^{\left(2\right)}\left(\theta ,\theta_{0}\right):=\log\left[\frac{\theta_{0}}{\theta }\right]$. Therefore following the proof of Proposition 8,

$$\begin{array}{cc} \int |f_{1}^{\left(2\right)}\left(\theta ,\theta_{0}\right){|}^{3}\rho_{n}\left(d\theta \right)=\int {\left|f_{3}^{\left(2\right)}\left(\theta ,\theta_{0}\right)\right|}^{3}\rho_{n}\left(d\theta \right)= & \int |f_{2}^{\left(1\right)}\left(\theta ,\theta_{0}\right){|}^{3}\rho_{n}\left(d\theta \right)<\frac{C_{2}}{n},\;\mathrm{and}\;,\\ \int |f_{2}^{\left(2\right)}\left(\theta ,\theta_{0}\right){|}^{3}\rho_{n}\left(d\theta \right)= & \int |f_{1}^{\left(1\right)}\left(\theta ,\theta_{0}\right){|}^{3}\rho_{n}\left(d\theta \right)<\frac{C_{1}}{n}. \end{array}$$

Finally, we take the KL-divergence $K(\rho_{n},\pi )$. $\rho_{n}$ follows a scaled Beta distribution on (0,1/2) with parameters $a_{n}=n\left(\theta_{0}/2\right)$ and $b_{n}=n\left(1-\theta_{0}/2\right)$, while π follows a scaled Beta distribution on (0,1/2) with parameters *a* and *b*. Thus,

$$\begin{array}{cc} K(\rho_{n},\pi ) & =\int_{0}^{\frac{1}{2}}\log\left(\frac{\rho_{n}\left(\theta \right)}{\pi (\theta )}\right)\rho_{n}\left(d\theta \right), \end{array}$$

which, by substituting t=2θ, we get,

$$\begin{array}{cc} K(\rho_{n},\pi ) & =2\int_{0}^{1}\log\left(\frac{\rho_{n}\left(t\right)}{\pi (t)}\right)\rho_{n}\left(dt\right). \end{array}$$

$\int_{0}^{1}\log\left(\frac{\rho_{n}\left(t\right)}{\pi (t)}\right)\rho_{n}\left(dt\right)$ is the KL-divergence between a Beta distribution with parameters $a_{n}$ and $b_{n}$ and a Beta distribution with parameters *a* and *b*. An application of Proposition A.2.1 gives us for a constant $C_{1}>0$,

$$\begin{array}{c} \int_{0}^{1}\log\left(\frac{\rho_{n}\left(t\right)}{\pi (t)}\right)\rho_{n}\left(dt\right)<C_{1}+\frac{1}{2}\log\left(n\right). \end{array}$$

Hence we can say, $K\left(\rho_{n},\pi \right)<2\left[C_{1}+\frac{1}{2}\log\left(n\right)\right]$. Thus, we now get that for some constant $C_{3}>0$,

$$\begin{array}{c} K\left(\rho_{n},\pi \right)<C_{3}\sqrt{n}. \end{array}$$

Choosing $C=\max(C_{1},C_{2},C_{3})$ we satisfy all of the conditions of Assumption 1 and thus by Theorem 3, we are complete the proof. □

#### Appendix B.3.4. Proof of Theorem 4

*Part 1: Verifying condition (i) of Corollary 1* As in the proof of Theorem 2 substitute the true parameter $\theta_{0}$ for $\theta_{1}$ and θ for $\theta_{2}$. We also set our initial distributions $q_{1}^{\left(0\right)}$ and $q_{2}^{\left(0\right)}$ to the known initial distribution $q^{\left(0\right)}$. A method similar to Equation (A35), yields

$$\begin{array}{cc} K(P_{\theta_{0}}^{\left(n\right)},P_{\theta }^{\left(n\right)}) & \leq \sum_{i=1}^{n}\sum_{k=1}^{m}E\left[M_{k}^{\left(1\right)}\left(X_{i},X_{i-1}\right)\right]\left|f_{k}^{\left(1\right)}\left(\theta ,\theta_{0}\right)\right|. \end{array}$$

Because $M_{k}^{\left(1\right)}$s satisfy Assumption 2, it follows by the application of Theorem 2.3, [21] that ∃λ>0 such that for any 0<κ≤λ, and for some ζ∈(0,1) possibly depending upon λ,

$$\begin{array}{c} E\left[e^{\kappa M_{k}^{\left(1\right)}\left(X_{i},X_{i-1}\right)}\right|X_{1},X_{0}]\leq \zeta^{i-1}e^{\kappa M_{k}^{\left(1\right)}\left(X_{1},X_{0}\right)}+\frac{1-\zeta^{i}}{1-\zeta }De^{\kappa a}\;\;\mathrm{for}\;\mathrm{all}\;i>1. \end{array}$$

We rewrite $E\left[M_{k}^{\left(1\right)}\left(X_{i},X_{i-1}\right)|X_{1},X_{0}\right]$ as follows:

$$\begin{array}{cc} E\left[M_{k}^{\left(1\right)}\left(X_{i},X_{i-1}\right)|X_{1},X_{0}\right] & =\frac{E[\kappa M_{k}^{\left(1\right)}\left(X_{i},X_{i-1}\right)|X_{1},X_{0}]}{\kappa }\\ \; & \leq \frac{E[e^{\kappa M_{k}^{\left(1\right)}\left(X_{i},X_{i-1}\right)}|X_{1},X_{0}]}{\kappa }. \end{array}$$

Therefore, $\sum_{i=1}^{n}E\left[M_{k}^{\left(1\right)}\left(X_{i},X_{i-1}\right)\right]$ can be upper bounded as,

$$\begin{array}{cc} \sum_{i=1}^{n}E\left[M_{k}^{\left(1\right)}\left(X_{i},X_{i-1}\right)\right] & =\sum_{i=1}^{n}E\left[\kappa M_{k}^{\left(1\right)}\left(X_{i},X_{i-1}\right)|X_{1},X_{0}\right]\kappa^{-1}\\ \; & \leq \sum_{i=1}^{n}\left[\zeta^{i-1}Ee^{\kappa M_{k}^{\left(1\right)}\left(X_{1},X_{0}\right)}+\frac{1-\zeta^{i}}{1-\zeta }De^{\kappa a}\right]\kappa^{-1}. \end{array}$$

Since, ζ∈(0,1), $\zeta^{i}<1$. Hence, we can write that,

$$\begin{array}{cc} \sum_{i=1}^{n}\left[\zeta^{i-1}Ee^{\kappa M_{k}^{\left(1\right)}\left(X_{1},X_{0}\right)}+\frac{1-\zeta^{i}}{1-\zeta }De^{\kappa a}\right]\kappa^{-1} & \leq \sum_{i=1}^{n}\left[\zeta^{i-1}Ee^{\kappa M_{k}^{\left(1\right)}\left(X_{1},X_{0}\right)}+\frac{1}{1-\zeta }De^{\kappa a}\right]\kappa^{-1}\\ \; & =\left[\frac{1-\zeta^{n}}{1-\zeta }Ee^{\kappa M_{k}^{\left(1\right)}\left(X_{1},X_{0}\right)}+\frac{n}{1-\zeta }De^{\kappa a}\right]\kappa^{-1}\\ \; & \leq nL, \end{array}$$

for a large constant *L*. Therefore $\int K\left(P_{\theta_{0}}^{\left(n\right)},P_{\theta }^{\left(n\right)}\right)\rho_{n}\left(d\theta \right)$ can be upper bounded as follows,

$$\begin{array}{cc} \int K\left(P_{\theta_{0}}^{\left(n\right)},P_{\theta }^{\left(n\right)}\right)\rho_{n}\left(d\theta \right) & \leq \int \sum_{k=1}^{m}nL\left|f_{k}^{\left(1\right)}\left(\theta ,\theta_{0}\right)\right|\rho_{n}\left(d\theta \right)\\ \; & =\sum_{k=1}^{m}nL\int \left|f_{k}^{\left(1\right)}\left(\theta ,\theta_{0}\right)\right|\rho_{n}\left(d\theta \right). \end{array}$$

By Assumption 1, $\int |f_{k}^{\left(1\right)}\left(\theta ,\theta_{0}\right)|\rho_{n}\left(d\theta \right)<\frac{C}{n}$, hence,

$$\begin{array}{cc} \int K\left(P_{\theta_{0}}^{\left(n\right)},P_{\theta }^{\left(n\right)}\right)\rho_{n}\left(d\theta \right) & \leq nL\frac{C}{\sqrt{n}}. \end{array}$$

Hence, for some $\epsilon_{n}^{\left(1\right)}=O\left(\frac{1}{\sqrt{n}}\right)$, we have obtained that, $\int K\left(P_{\theta_{0}}^{\left(n\right)},P_{\theta }^{\left(n\right)}\right)\rho_{n}\left(d\theta \right)\leq n\epsilon_{n}^{\left(1\right)}$.

*Part 2: Verifying condition (ii) of Corollary 1:* Similar to as in the proof of Theorem 3, we upper bound $\int \mathrm{Var}\left[r_{n}\left(\theta ,\theta_{0}\right)\right]\rho_{n}\left(d\theta \right)$ by

(A39) $$\begin{array}{cc} \int \mathrm{Var}\left[r_{n}\left(\theta ,\theta_{0}\right)\right]\rho_{n}\left(d\theta \right) & \leq \sum_{i,j=1}^{n}(\frac{4}{n}+2n^{\delta /2}(\int C_{\theta_{0},\theta }^{\left(i\right)}\rho_{n}\left(d\theta \right)+\int C_{\theta_{0},\theta }^{\left(j\right)}\rho_{n}\left(d\theta \right) \end{array}$$

(A40) $$\begin{array}{cc} \; & \;\;+\int \sqrt{C_{\theta_{0},\theta }^{\left(i\right)}C_{\theta_{0},\theta }^{\left(j\right)}}\rho_{n}\left(d\theta \right)))\left(\alpha_{|i-j|-1}^{\delta /(2+\delta )}\right)\\ \; & \;+\sum_{i=1}^{n}\left(\frac{4}{n}+2n^{\delta /2}\int C_{\theta_{0},\theta }^{\left(i\right)}\rho_{n}\left(d\theta \right)\right)\left(\alpha_{i-1}^{\delta /(2+\delta )}\right), \end{array}$$

where $C_{\theta_{0},\theta }$ is upper bounded as

$$\begin{array}{cc} C_{\theta_{0},\theta }^{\left(i\right)} & \leq \sum_{k=1}^{m}m^{1+\delta }E{\left[M_{k}^{\left(1\right)}\left(X_{i},X_{i-1}\right)\right]}^{2+\delta }{\left|f_{k}^{\left(1\right)}\left(\theta ,\theta_{0}\right)\right|}^{2+\delta }. \end{array}$$

There exists a constant $C_{\delta }$ depending upon δ such that,

$$\begin{array}{cc} {\left[M_{k}^{\left(1\right)}\right]}^{2+\delta }\left(X_{i},X_{i-1}\right) & =\frac{\kappa^{2+\delta }{\left[M_{k}^{\left(1\right)}\right]}^{2+\delta }{\left(X_{i},X_{i-1}\right)}^{2+\delta }}{\kappa^{2+\delta }}\\ \; & \leq \frac{e^{\kappa M_{k}^{\left(1\right)}\left(X_{i},X_{i-1}\right)}+C_{\delta }}{\kappa^{2+\delta }}. \end{array}$$

By expressing $E\left[M_{k}^{\left(1\right)}{\left(X_{i},X_{i-1}\right)}^{2+\delta }\right]=E\left[E\left[M_{k}^{\left(1\right)}{\left(X_{i},X_{i-1}\right)}^{2+\delta }|X_{1},X_{0}\right]\right]$ and following a method similar to the previous part, we get,

$$\begin{array}{cc} E\left[M_{k}^{\left(1\right)}{\left(X_{i},X_{i-1}\right)}^{2+\delta }\right] & \leq \frac{\left[\zeta^{i}Ee^{\kappa M_{k}^{\left(1\right)}\left(X_{1},X_{0}\right)}+\frac{1-\zeta^{i}}{1-\zeta }De^{\kappa a}\right]+C_{\delta }}{\kappa^{2+\delta }}. \end{array}$$

The fact that 0<ζ<1 implies that $0<\zeta^{i}<\zeta$. This gives us the following,

$$\begin{array}{cc} E\left[M_{k}^{\left(1\right)}{\left(X_{i},X_{i-1}\right)}^{2+\delta }\right] & \leq \frac{\left[\zeta Ee^{\kappa M_{k}^{\left(1\right)}\left(X_{1},X_{0}\right)}+\frac{1}{1-\zeta }De^{\kappa a}\right]+C_{\delta }}{\kappa^{2+\delta }}. \end{array}$$

Since κ<λ, by the application of Jensen’s inequality, we get

$$\begin{array}{cc} E\left[M_{k}^{\left(1\right)}{\left(X_{i},X_{i-1}\right)}^{2+\delta }\right] & \leq \frac{\left[\zeta Ee^{\lambda M_{k}^{\left(1\right)}\left(X_{1},X_{0}\right)}+\frac{1}{1-\zeta }De^{\kappa a}\right]+C_{\delta }}{\kappa^{2+\delta }}\\ \; & =\frac{\left[\zeta \int e^{\lambda M_{k}^{\left(1\right)}\left(x_{1},x_{0}\right)}p_{\theta_{0}}\left(x_{1}|x_{0}\right)D\left(x_{0}\right)dx_{1}dx_{0}+\frac{1}{1-\zeta }De^{\kappa a}\right]+C_{\delta }}{\kappa^{2+\delta }}. \end{array}$$

We know that $\int |f_{k}^{\left(1\right)}\left(\theta ,\theta_{0}\right){|}^{2+\delta }\rho_{n}\left(d\theta \right)<\frac{C}{n}$. Thus, following Assumption 1 we can say that, for a large constant *L*, $\int C_{\theta_{0},\theta }^{\left(i\right)}\rho_{n}\left(d\theta \right)\leq \frac{L}{n}$. The rest of the proof follows similarly as in the proof of Theorem 3, and we obtain an $\epsilon_{n}^{\left(2\right)}=O\left(\frac{n^{\delta /2}}{n}\right)$, such that,

$$\begin{array}{c} \int \mathrm{Var}\left[r_{n}\left(\theta ,\theta_{0}\right)\right]\rho_{n}\left(d\theta \right)<n\epsilon_{n}^{\left(2\right)}. \end{array}$$

Since, $K\left(\rho_{n},\pi \right)\leq \sqrt{n}C$, similar arguments as in the proof of Theorem 2 holds. The theorem is thus proved.

#### Appendix B.3.5. Proof of Theorem 5

*Part 1: Verifying condition (i) of Corollary 1* As in the proof of Theorem 2 substitute the true parameter $\theta_{0}$ for $\theta_{1}$ and θ for $\theta_{2}$. We also set $q_{1}^{\left(0\right)}$ and $q_{2}^{\left(0\right)}$ to the known initial distribution $q^{\left(0\right)}$. Similar to the steps leading to Equation (A35), we get

$$\begin{array}{cc} K(P_{\theta_{0}}^{\left(n\right)},P_{\theta }^{\left(n\right)}) & \leq \sum_{i=1}^{n}\sum_{k=1}^{m}E\left[M_{k}^{\left(1\right)}\left(X_{i},X_{i-1}\right)\right]\left|f_{k}^{\left(1\right)}\left(\theta ,\theta_{0}\right)\right|. \end{array}$$

Consider the term $E\left[M_{k}^{\left(1\right)}\left(X_{i},X_{i-1}\right)\right]$. With $q_{\theta_{0}}^{\left(i-1\right)}$ the marginal distribution of $X_{i-1}$, we have

$$\begin{array}{cc} E\left[M_{k}^{\left(1\right)}\left(X_{i},X_{i-1}\right)\right] & =\int M_{k}^{\left(1\right)}\left(x_{i},x_{i-1}\right)p_{\theta_{0}}\left(x_{i}|x_{i-1}\right)q_{\theta_{0}}^{\left(i-1\right)}\left(x_{i-1}\right)dx_{i}dx_{i-1}.\\ E\left[M_{k}^{\left(1\right)}\left(X_{i},X_{i-1}\right)\right] & =\int M_{k}^{\left(1\right)}\left(x_{i},x_{i-1}\right)p_{\theta_{0}}\left(x_{i}|x_{i-1}\right)p_{\theta_{0}}^{i-1}\left(x_{i-1}|x_{0}\right)q_{\theta_{0}}^{\left(0\right)}\left(x_{0}\right)dx_{0}dx_{i}dx_{i-1} \end{array}$$

Recall that the marginal density satisfies $q_{\theta_{0}}^{\left(i-1\right)}\left(x_{i-1}\right)=\int p_{\theta_{0}}^{i-1}\left(x_{i-1}|x_{0}\right)q_{\theta_{0}}^{\left(0\right)}\left(x_{0}\right)d\left(x_{0}\right)$, where $p_{\theta_{0}}^{i}\left(\cdot |x_{0}\right)$ is the *i*-step transition probability. Then

$$\begin{array}{cc} E\left[M_{k}^{\left(1\right)}\left(X_{i},X_{i-1}\right)\right] & =\int E\left[M_{k}^{\left(1\right)}\left(X_{i},x_{i-1}\right)|x_{i-1}\right]p_{\theta_{0}}^{i-1}\left(x_{i-1}|x_{0}\right)q_{\theta_{0}}^{\left(0\right)}\left(x_{0}\right)dx_{0}dx_{i-1}. \end{array}$$

Since the Markov chain $\left\{X_{n}\right\}$ satisfies Assumption A.1.1, we know by the application of Theorem A.1.1 that $\left\{X_{n}\right\}$ is *V*-geometrically ergodic. Hence, ∃τ<1, R<∞ such that ∀|f|<V

$$\begin{array}{cc} |\int f\left(x_{i-1}\right)p_{\theta_{0}}^{i-1}\left(x_{i-1}|x_{0}\right)dx_{i-1} & -\int f\left(x_{i-1}\right)q_{\theta_{0}}\left(x_{i-1}\right)dx_{i-1}|<RV\left(x_{0}\right)\tau^{i-1}, \end{array}$$

where $q_{\theta_{0}}$ is the stationary distribution, implying that

$$\begin{array}{cc} \int f\left(x_{i-1}\right)p_{\theta_{0}}^{i-1}\left(x_{i-1}|x_{0}\right)dx_{i-1} & <\int f\left(x_{i-1}\right)q_{\theta_{0}}\left(x_{i-1}\right)dx_{i-1}+RV\left(x_{0}\right)\tau^{i-1}. \end{array}$$

By the application of Jensen’s inequality we get ${\left(E\left[M_{k}^{\left(1\right)}\left(X_{i},X_{i-1}\right)|X_{i-1}\right]\right)}^{2+\delta }\leq E\left[M_{k}^{\left(1\right)}{\left(X_{i},X_{i-1}\right)}^{2+\delta }|X_{i-1}\right]<V\left(X_{i-1}\right)$. Since V(·)≥1, it follows from the previous expression that $E\left[M_{k}^{\left(1\right)}\left(X_{i},X_{i-1}\right)|X_{i-1}\right]<V{\left(X_{i-1}\right)}^{1/(2+\delta )}\leq V\left(X_{i-1}\right)$. Thus, setting $f\left(x\right)=E\left[M_{k}^{\left(1\right)}\left(X_{i},X_{i-1}\right)|X_{i-1}=x\right]$, we obtain

$$\begin{array}{cc} E\left[M_{k}^{\left(1\right)}\left(X_{i},X_{i-1}\right)\right] & <\int \left[E\left[M_{k}^{\left(1\right)}\left(X_{i},X_{i-1}\right)|X_{i-1}\right]q_{\theta_{0}}\left(x_{i}\right)dx_{i-1}+RV\left(x_{0}\right)\tau^{i-1}\right]q^{\left(0\right)}\left(x_{0}\right)dx_{0}\\ \; & =E\left[M_{k}^{\left(1\right)}\left(X_{1},X_{0}\right)\right]+\tau^{i-1}\int RV\left(x_{0}\right)q^{\left(0\right)}\left(x_{0}\right)dx_{0}. \end{array}$$

Summing from i=1 to *n*, we get

$$\begin{array}{cc} \sum_{i=1}^{n}E\left[M_{k}^{\left(1\right)}\left(x_{i},x_{i-1}\right)\right] & <nE\left[M_{k}^{\left(1\right)}\left(X_{1},X_{0}\right)\right]+\sum_{i=1}^{n}\tau^{i-1}\int RV\left(x_{0}\right)q^{\left(0\right)}\left(x_{0}\right)dx_{0} \end{array}$$

$$\begin{array}{cc} \; & =nE\left[M_{k}^{\left(1\right)}\left(X_{1},X_{0}\right)\right]+\frac{1-\tau^{n}}{1-\tau }\int RV\left(x_{0}\right)q^{\left(0\right)}\left(x_{0}\right)dx_{0}. \end{array}$$

This gives us the following bound on $\int K\left(P_{\theta_{0}}^{\left(n\right)},P_{\theta }^{\left(n\right)}\right)\rho_{n}\left(d\theta \right)$:

$$\begin{array}{cc} \int K\left(P_{\theta_{0}}^{\left(n\right)},P_{\theta }^{\left(n\right)}\right)\rho_{n}\left(d\theta \right) & \leq \sum_{k=1}^{m}\left[nE\left[M_{k}^{\left(1\right)}\left(X_{1},X_{0}\right)\right]+\frac{1-\tau^{n}}{1-\tau }\int RV\left(x_{0}\right)D\left(x_{0}\right)dx_{0}\right]\\ \; & \;\times \int |f_{k}^{\left(1\right)}\left(\theta ,\theta_{0}\right)|\rho_{n}\left(d\theta \right). \end{array}$$

By Assumption 1, $\int |f_{k}^{\left(1\right)}\left(\theta ,\theta_{0}\right)|\rho_{n}\left(d\theta \right)<\frac{C}{\sqrt{n}}$. Hence, we can rewrite the previous expression as

$$\begin{array}{cc} \int K\left(P_{\theta_{0}}^{\left(n\right)},P_{\theta }^{\left(n\right)}\right)\rho_{n}\left(d\theta \right) & \leq \sum_{k=1}^{m}\left[nE\left[M_{k}^{\left(1\right)}\left(X_{1},X_{0}\right)\right]+\frac{1-\tau^{n}}{1-\tau }\int RV\left(x_{1}\right)D\left(x_{1}\right)dx_{1}\right]\frac{C}{\sqrt{n}}\\ \; & =nm\left[E\left[M_{k}^{\left(1\right)}\left(X_{1},X_{0}\right)\right]+\frac{1-\tau^{n}}{n(1-\tau )}\int RV\left(x_{0}\right)D\left(x_{0}\right)dx_{0}\right]\frac{C}{\sqrt{n}}. \end{array}$$

Since, τ<1, $0<1-\tau^{n}<1$, and we rewrite the previous equation as,

$$\begin{array}{cc} \int K\left(P_{\theta_{0}}^{\left(n\right)},P_{\theta }^{\left(n\right)}\right)\rho_{n}\left(d\theta \right) & \leq nm\left[E\left[M_{k}^{\left(1\right)}\left(X_{1},X_{0}\right)\right]+\frac{1}{n(1-\tau )}\int RV\left(x_{0}\right)D\left(x_{0}\right)dx_{0}\right]\frac{C}{\sqrt{n}}. \end{array}$$

Hence, there exists an $\epsilon_{n}^{\left(1\right)}=O\left(\frac{1}{\sqrt{n}}\right)$ such that $\int K\left(P_{\theta_{0}}^{\left(n\right)},P_{\theta }^{\left(n\right)}\right)\rho_{n}\left(d\theta \right)\leq n\epsilon_{n}^{\left(1\right)}$.

*Part 2: Verifying condition (ii) of Corollary 1:* Similar to as in the proof of Theorem 3, we upper bound $\int \mathrm{Var}\left[r_{n}\left(\theta ,\theta_{0}\right)\right]\rho_{n}\left(d\theta \right)$ by

(A41) $$\begin{array}{cc} \int \mathrm{Var}\left[r_{n}\left(\theta ,\theta_{0}\right)\right]\rho_{n}\left(d\theta \right) & \leq \sum_{i,j=1}^{n}(\frac{4}{n}+2n^{\delta /2}(\int C_{\theta_{0},\theta }^{\left(i\right)}\rho_{n}\left(d\theta \right)+\int C_{\theta_{0},\theta }^{\left(j\right)}\rho_{n}\left(d\theta \right) \end{array}$$

(A42) $$\begin{array}{cc} \; & \;+\int \sqrt{C_{\theta_{0},\theta }^{\left(i\right)}C_{\theta_{0},\theta }^{\left(j\right)}}\rho_{n}\left(d\theta \right)))\left(\alpha_{|i-j|-1}^{\delta /(2+\delta )}\right)\\ \; & \;+\sum_{i=1}^{n}\left(\frac{4}{n}+2n^{\delta /2}\int C_{\theta_{0},\theta }^{\left(i\right)}\rho_{n}\left(d\theta \right)\right)\left(\alpha_{i-1}^{\delta /(2+\delta )}\right), \end{array}$$

where $C_{\theta_{0},\theta }$ is upper bounded as

$$\begin{array}{cc} C_{\theta_{0},\theta }^{\left(i\right)} & \leq \sum_{k=1}^{m}m^{1+\delta }E{\left[M_{k}^{\left(1\right)}\left(X_{i},X_{i-1}\right)\right]}^{2+\delta }{\left|f_{k}^{\left(1\right)}\left(\theta ,\theta_{0}\right)\right|}^{2+\delta }. \end{array}$$

Since $E\left[M_{k}^{\left(1\right)}{\left(X_{i},X_{i-1}\right)}^{2+\delta }|X_{i-1}\right]<V\left(X_{i-1}\right)$, by a similar application of *V*-geometric ergodicity, we can say that, ∃0<τ<1, such that

$$\begin{array}{cc} E{\left[M_{k}^{\left(1\right)}\left(X_{i},X_{i-1}\right)\right]}^{2+\delta } & \leq nE{\left[M_{k}^{\left(1\right)}\left(X_{1},X_{0}\right)\right]}^{2+\delta }+\tau^{i-1}\int RV\left(x_{0}\right)D\left(x_{0}\right)dx_{0}, \end{array}$$

which, by the fact that $\tau^{i-1}<\tau$, gives us,

$$\begin{array}{cc} E{\left[M_{k}^{\left(1\right)}\left(X_{i},X_{i-1}\right)\right]}^{2+\delta } & \leq E{\left[M_{k}^{\left(1\right)}\left(X_{1},X_{0}\right)\right]}^{2+\delta }+\tau \int RV\left(x_{0}\right)D\left(x_{0}\right)dx_{0}. \end{array}$$

By Assumption 1, we know that, $\int |f_{k}^{\left(1\right)}\left(\theta ,\theta_{0}\right){|}^{2+\delta }\rho_{n}\left(d\theta \right)<\frac{C}{n}$. Hence, for a large constant *L*, $\int C_{\theta_{0},\theta }^{\left(i\right)}\rho_{n}\left(d\theta \right)\leq \frac{L}{n}$. We also see that since the chain is geometrically ergodic, by the application of Equation (A4), $\sum_{k\geq 1}\alpha_{k}^{\delta /(2+\delta )}<+\infty$. The rest of the proof follows similarly as in the proof of Theorem 3, and we obtain an $\epsilon_{n}^{\left(2\right)}=O\left(\frac{n^{\delta /2}}{n}\right)$, such that,

$$\begin{array}{c} \int \mathrm{Var}\left[r_{n}\left(\theta ,\theta_{0}\right)\right]\rho_{n}\left(d\theta \right)<n\epsilon_{n}^{\left(2\right)}. \end{array}$$

Since, $K\left(\rho_{n},\pi \right)\leq \sqrt{n}C$, similar arguments as in the proof of Theorem 2 holds. The theorem is thus proved. □

#### Appendix B.3.6. Proof of Proposition 10

For the purpose of the proof, we choose $\rho_{n}$’s with scaled Beta distribution with parameters $a_{n}=n\frac{1+\theta_{0}}{2}$ and $b_{n}=n\frac{1-\theta_{0}}{2}$. Since, $\rho_{n}$ is a scaled Beta distribution with the scaling factors m=2 and c=−1, the pdf of $\rho_{n}$ is given by

$$\begin{array}{cc} \rho_{n}\left(\theta \right) & =\frac{1}{2\mathrm{Beta}(a_{n},b_{n})}{\left(\frac{1+\theta }{2}\right)}^{a_{n}}{\left(\frac{1-\theta }{2}\right)}^{b_{n}} \end{array}$$

Since this is a scaled distribution, $E_{\rho_{n}}\left[\theta \right]=2\frac{a_{n}}{a_{n}+b_{n}}-1=\theta_{0}$ and there exists a constant σ>0, ${\mathrm{Var}}_{\rho_{n}}\left[\theta \right]=\frac{\sigma^{2}}{n}$. We now analyse the log-ratio of the transition probabilities for the Markov chain,

$$\begin{array}{c} \log p_{\theta_{0}}\left(X_{n}|X_{n-1}\right)-\log p_{\theta }\left(X_{n}|X_{n-1}\right)=2X_{n}X_{n-1}\left(\theta -\theta_{0}\right)+X_{n-1}^{2}\left(\theta_{0}^{2}-\theta^{2}\right). \end{array}$$

Observe that in this setting, $M_{1}^{\left(1\right)}\left(X_{n},X_{n-1}\right)=\left|X_{n}X_{n-1}\right|$ and $M_{2}^{\left(1\right)}\left(X_{n},X_{n-1}\right)=X_{n}^{2}$. Next, using the fact that

$$\begin{array}{cc} E[|X_{n}{|}^{2+\delta }|X_{n-1}] & =E[|X_{n}-\theta_{0}X_{n-1}+\theta_{0}X_{n-1}{|}^{2+\delta }|X_{n-1}], \end{array}$$

and by an application of triangle inequality, we obtain

$$\begin{array}{cc} E[|X_{n}{|}^{2+\delta }|X_{n-1}] & \leq E\left[{\left(|X_{n}-\theta_{0}X_{n-1}\left|+\right|\theta_{0}X_{n-1}|\right)}^{2+\delta }|X_{n-1}\right]\\ \; & =E\left[{\left(2\frac{|X_{n}-\theta_{0}X_{n-1}\left|+\right|\theta_{0}X_{n-1}|}{2}\right)}^{2+\delta }|X_{n-1}\right]\\ \; & =E\left[2^{2+\delta }{\left(\frac{|X_{n}-\theta_{0}X_{n-1}\left|+\right|\theta_{0}X_{n-1}|}{2}\right)}^{2+\delta }|X_{n-1}\right]. \end{array}$$

Now by using Jensen’s inequality we get,

$$\begin{array}{cc} E[|X_{n}{|}^{2+\delta }|X_{n-1}] & \leq E\left[2^{2+\delta }\left(\frac{|X_{n}-\theta_{0}X_{n-1}{|}^{2+\delta }+{\left|\theta_{0}X_{n-1}\right|}^{2+\delta }}{2}\right)|X_{n-1}\right]\\ \; & =2^{1+\delta }E\left[|X_{n}-\theta_{0}X_{n-1}{|}^{2+\delta }|X_{n-1}\right]+2^{1+\delta }\left|\theta_{0}X_{n-1}\right|. \end{array}$$

We know if $Y∼N(\mu ,\sigma^{2})$, then ${E|Y-\mu |}^{p}=\sigma^{p}\frac{2^{\frac{p}{2}\Gamma \left(\frac{p+1}{2}\right)}}{\sqrt{\pi }}$. Consequently,

(A43) $$\begin{array}{cc} \;E[|X_{n}{|}^{2+\delta }|X_{n-1}] & \leq 2^{1+\delta }\left[\frac{2^{\frac{2+\delta }{2}\Gamma \left(\frac{3+\delta }{2}\right)}}{\sqrt{\pi }}\right]+2^{1+\delta }{\left|\theta_{0}X_{n-1}\right|}^{2+\delta }. \end{array}$$

It follows that,

$$\begin{array}{cc} E[M_{1}^{\left(1\right)}{\left(X_{n},X_{n-1}\right)}^{2+\delta }|X_{n-1}] & \leq 2^{1+\delta }\left[\frac{2^{\frac{2+\delta }{2}\Gamma \left(\frac{3+\delta }{2}\right)}}{\sqrt{\pi }}\right]|X_{n-1}{|}^{2+\delta }+2^{1+\delta }|\theta_{0}{|}^{2+\delta }{\left|X_{n-1}\right|}^{4+2\delta }\\ \; & \leq \left(2^{1+\delta }\left[\frac{2^{\frac{2+\delta }{2}\Gamma \left(\frac{3+\delta }{2}\right)}}{\sqrt{\pi }}\right]+2^{1+\delta }{\left|\theta_{0}\right|}^{2+\delta }\right)\left(\right|X_{n-1}{|}^{4+2\delta }+1). \end{array}$$

Since $\theta_{0}<1$, we can say,

$$\begin{array}{cc} E[M_{1}^{\left(1\right)}{\left(X_{n},X_{n-1}\right)}^{2+\delta }|X_{n-1}] & \leq \left(2^{1+\delta }\left[\frac{2^{\frac{2+\delta }{2}\Gamma \left(\frac{3+\delta }{2}\right)}}{\sqrt{\pi }}\right]+2^{1+\delta }\right)\left(\right|X_{n-1}{|}^{4+2\delta }+1). \end{array}$$

Define a constant $C_{\delta }:=\left(2^{1+\delta }\left[\frac{2^{\frac{2+\delta }{2}\Gamma \left(\frac{3+\delta }{2}\right)}}{\sqrt{\pi }}\right]+2^{1+\delta }\right)$. The above term then becomes,

$$\begin{array}{cc} E[M_{1}^{\left(1\right)}{\left(X_{n},X_{n-1}\right)}^{2+\delta }|X_{n-1}] & \leq C_{\delta }\left(\right|X_{n-1}{|}^{4+2\delta }+1). \end{array}$$

Next we analyse the term $M_{2}^{\left(1\right)}\left(X_{n},X_{n-1}\right)$.

$$\begin{array}{cc} E\left[M_{2}^{\left(1\right)}{\left(X_{n},X_{n-1}\right)}^{2+\delta }|X_{n-1}\right] & =E[X_{n-1}^{4+2\delta }|X_{n-1}]\\ \; & =X_{n-1}^{4+2\delta }\\ \; & \leq C_{\delta }\left(X_{n-1}^{4+2\delta }+1\right). \end{array}$$

Then, defining $V\left(x\right):=C_{\delta }\left(x^{4+2\delta }+1\right)$ it follows that,

$$\begin{array}{cc} E\left[V\left(X_{n}\right)|X_{n-1}\right] & =E\left[C_{\delta }\left(X_{n}^{4+2\delta }+1\right)|X_{n-1}\right]. \end{array}$$

Using a technique similar to Equation (A43) we get,

$$\begin{array}{cc} E\left[C_{\delta }\left(X_{n}^{4+2\delta }+1\right)|X_{n-1}\right] & \leq \left[C_{\delta }(2^{3+2\delta }\left[\frac{2^{\frac{4+2\delta }{2}\Gamma \left(\frac{5+2\delta }{2}\right)}}{\sqrt{\pi }}\right]+2^{3+2\delta }|\theta_{0}X_{n-1}{|}^{4+2\delta }+1)\right]. \end{array}$$

Define another constant $C_{\delta }^{\prime }:=C_{\delta }\left(2^{3+2\delta }\left[\frac{2^{\frac{4+2\delta }{2}\Gamma \left(\frac{5+2\delta }{2}\right)}}{\sqrt{\pi }}\right]-2^{3+2\delta }{\left|\theta_{0}\right|}^{4+2\delta }+1\right)$. Since δ>0, $\frac{2^{\frac{4+2\delta }{2}\Gamma \left(\frac{5+2\delta }{2}\right)}}{\sqrt{\pi }}>1$. Furthermore, since $|\theta_{0}|<1$, so is $|\theta_{0}{|}^{4+2\delta }$. Hence,

$$2^{3+2\delta }\left[\frac{2^{\frac{4+2\delta }{2}\Gamma \left(\frac{5+2\delta }{2}\right)}}{\sqrt{\pi }}\right]-2^{3+2\delta }{\left|\theta_{0}\right|}^{4+2\delta }>0.$$

Hence, we have shown that,

$$\begin{array}{cc} E\left[V\left(X_{n}\right)|X_{n-1}\right] & \leq (2^{3+2\delta }|\theta_{0}{|}^{4+2\delta })C_{\delta }\left(X_{n-1}^{4+2\delta }+1\right)+C_{\delta }^{\prime }. \end{array}$$

Since $|\theta_{0}|<2^{\frac{1}{4+2\delta }-1}$, $2^{3+2\delta }{\left|\theta_{0}\right|}^{4+2\delta }<1$, and we can express the above equation as,

$$\begin{array}{cc} E\left[V\left(X_{n}\right)|X_{n-1}\right] & \leq V\left(X_{n-1}\right)+C_{\delta }^{\prime }. \end{array}$$

Define the set $C\left(m\right):=\{x:|x{|}^{4+2\delta }+1\leq m\}$. From Proposition 11.4.2, [20], for a large enough *m*, C(m) forms a petite set. Thus, we have proved that V(x) as defined in this example satisfies Assumption A.1.1, and $\left\{X_{n}\right\}$ is *V*-geometrically ergodic. The $f_{j}^{\left(1\right)}$’s corresponding to Assumption 1 are given by $f_{1}^{\left(1\right)}\left(\theta ,\theta_{0}\right)=\left(\theta -\theta_{0}\right)$ and $f_{2}^{\left(1\right)}\left(\theta ,\theta_{0}\right)=\left(\theta_{0}^{2}-\theta^{2}\right)$. Therefore, it follows that,

$$\begin{array}{cc} \partial_{\theta }f_{1}^{\left(1\right)} & =1,\\ \partial_{\theta }f_{2}^{\left(1\right)} & =-2\theta \;\mathrm{and}\;\\ -2 & <-2\theta <2. \end{array}$$

Since $f_{1}^{\left(1\right)}\left(\theta_{0},\theta_{0}\right)=f_{2}^{\left(1\right)}\left(\theta_{0},\theta_{0}\right)=0$, We just showed that they also have bounded partial derivatives. We also know that |θ|<1. Hence, by Proposition 4$f_{j}^{\left(1\right)}$’s satisfy the conditions of Assumption 1.

The invariant distribution for the simple linear model Markov-chain under parameter θ is given by a gaussian distribution with mean 0 and variance $\frac{1}{1-\theta^{2}}$. In other words,

$$\begin{array}{c} q_{\theta }\left(x\right)=\frac{1}{\sqrt{2\pi }}e^{-\frac{1-\theta^{2}}{2}x^{2}}. \end{array}$$

Analyzing the log likelihood yields,

$$\begin{array}{cc} \log q_{0}\left(x\right)-\log q_{\theta }\left(x\right) & =-\frac{x^{2}}{2}\left(1-\theta_{0}^{2}\right)+\frac{x^{2}}{2}\left(1-\theta^{2}\right)\\ \; & =\frac{x^{2}}{2}\left(\theta_{0}^{2}-\theta^{2}\right). \end{array}$$

Let $f_{1}^{\left(2\right)}\left(\theta_{0},\theta_{0}\right)=\left(\theta_{0}^{2}-\theta^{2}\right)$ and $f_{1}^{\left(2\right)}\left(\theta_{0},\theta_{0}\right)=0$. Since $f_{1}^{\left(2\right)}\left(\theta_{0},\theta_{0}\right)=f_{2}^{\left(1\right)}\left(\theta_{0},\theta_{0}\right)$, by following arguments similar as before, can conclude that $f_{1}^{\left(2\right)}\left(\theta_{0},\theta_{0}\right)$ also satisfies the requirements of Assumption 1. Let $M_{1}^{\left(2\right)}\left(x\right)=\frac{x^{2}}{2}$ and define $M_{2}^{\left(2\right)}\left(x\right):=1$. Let $X_{0}∼q_{1}^{\left(0\right)}$. As long as $\int x^{4+2\delta }q_{1}^{\left(0\right)}\left(x\right)dx<\infty$, we satisfy all the conditions required for Theorem 5. Finally we need to verify the condition that $K\left(\rho_{n},\pi \right)<C\sqrt{n}$ for some constant C>0. The KL-divergence $\int \log\left(\frac{\rho_{n}\left(\theta \right)}{\pi (\theta )}\right)\rho_{n}\left(d\theta \right)$ becomes,

$$\begin{array}{cc} K(\rho_{n},\pi ) & =\int_{-1}^{1}\log\left(\frac{1}{2\mathrm{Beta}(a_{n},b_{n})}{\left(\frac{1+\theta }{2}\right)}^{a_{n}}{\left(\frac{1-\theta }{2}\right)}^{b_{n}}\right)\\ \; & \;\times \frac{1}{2\mathrm{Beta}(a_{n},b_{n})}{\left(\frac{1+\theta }{2}\right)}^{a_{n}}{\left(\frac{1-\theta }{2}\right)}^{b_{n}}d\theta . \end{array}$$

Substituting, $y=\frac{1+\theta }{2}$, we get,

$$\begin{array}{cc} K(\rho_{n},\pi ) & =\int_{0}^{1}\log\left(\frac{1}{2\mathrm{Beta}(a_{n},b_{n})}{\left(y\right)}^{a_{n}}{\left(1-y\right)}^{b_{n}}\right)\frac{1}{2\mathrm{Beta}(a_{n},b_{n})}{\left(y\right)}^{a_{n}}{\left(1-y\right)}^{b_{n}}dy\\ \; & =\int_{0}^{1}\log\left(\frac{1}{2}\right)\frac{1}{\mathrm{Beta}(a_{n},b_{n})}{\left(y\right)}^{a_{n}}{\left(1-y\right)}^{b_{n}}dy\\ \; & \;+\int_{0}^{1}\log\left(\frac{1}{\mathrm{Beta}(a_{n},b_{n})}{\left(y\right)}^{a_{n}}{\left(1-y\right)}^{b_{n}}\right)\frac{1}{\mathrm{Beta}(a_{n},b_{n})}{\left(y\right)}^{a_{n}}{\left(1-y\right)}^{b_{n}}. \end{array}$$

The first term integrates up to log(1/2). The second term is the KL-divergence between a Uniform and Beta distribution with parameters $a_{n}=n\frac{1+\theta_{0}}{2}$ and $b_{n}=n\left(1-\frac{1+\theta_{0}}{2}\right)$ and support [0,1]. Following Lemma A.2.1 it follows that $K(\rho_{n},\pi )$ is upper bounded by,

$$\begin{array}{cc} K(\rho_{n},\pi ) & <\log(1/2)+C_{1}+\frac{1}{2}\log\left(n\right)<C\sqrt{n}, \end{array}$$

for some large constant *C*. This completes the proof. □

### Appendix B.4. Proofs for Misspecified Models

#### Proof of Theorem 6

As in the proof of Theorem 1, following Equation (A13), we note that,

(A44) $$\begin{array}{cc} \;\int D_{\alpha^{re}}\left(P_{\theta }^{\left(n\right)},P_{\theta_{0}}^{\left(n\right)}\right){\tilde{\pi }}_{n,\alpha^{re}|X^{n}}\left(d\theta \right) & \leq \frac{\alpha^{re}}{1-\alpha^{re}}\int K\left(P_{\theta_{0}}^{\left(n\right)},P_{\theta }^{\left(n\right)}\right)\rho_{n}\left(d\theta \right)\\ & \;+\frac{\alpha^{re}}{1-\alpha^{re}}\sqrt{\frac{\mathrm{Var}[\int r_{n}\left(\theta ,\theta_{0}\right)\rho_{n}\left(d\theta \right)]}{\eta }}+\frac{K\left(\rho_{n},\pi \right)-\log\left(\epsilon \right)}{1-\alpha^{re}}. \end{array}$$

Following from Equations (23) and (26), we get that,

$$\begin{array}{cc} \int K\left(P_{\theta_{0}}^{\left(n\right)},P_{\theta }^{\left(n\right)}\right)\rho_{n}\left(d\theta \right) & \leq E\left[r_{n}\left(\theta_{0},\theta_{n}^{*}\right)\right]+n\epsilon_{n}, \end{array}$$

and

$$\begin{array}{cc} \int \mathrm{Var}\left[r_{n}\left(\theta ,\theta_{0}\right)\right]\rho_{n}\left(d\theta \right) & \leq 2n\epsilon_{n}+2\mathrm{Var}\left[r_{n}\left(\theta_{n}^{*},\theta_{0}\right)\right]. \end{array}$$

Plugging these into Equation (A44), we are done. □
